# Therapeutic targeting of P2X4 receptor and mitochondrial metabolism in clear cell renal carcinoma models

**DOI:** 10.1186/s13046-023-02713-1

**Published:** 2023-05-26

**Authors:** Christofer Rupert, Carmela Dell’ Aversana, Laura Mosca, Vittorino Montanaro, Davide Arcaniolo, Marco De Sio, Antonio Bilancio, Lucia Altucci, Wulf Palinski, Roberto Pili, Filomena de Nigris

**Affiliations:** 1 Division of Hematology and Oncology, Jacobs School of Medicine and Biomedical Sciences, University at Buffalo, Buffalo, NY USA; 2 Institute of Experimental Endocrinology and Oncology, Gaetano Salvatore (IEOS)-CNR, Naples, Italy; 3 Department of Precision Medicine, University of Campania L. Vanvitelli, Naples, Italy; 4 Urology Unit, San Leonardo Hospital, Castellammare Di Stabia, Naples, Italy; 5 Department of Women, Child, and General and Specialistic Surgery, University of Campania L. Vanvitelli, Naples, Italy; 6 BIOGEM, Ariano Irpino, Avellino, Italy; 7 Department of Medicine, University of California San Diego, La Jolla, CA USA

**Keywords:** Organoids, Mitochondria, Renal carcinoma, Lysosomes, Purinergic receptors, Drug screening

## Abstract

**Background:**

Clear cell renal cell carcinoma (ccRCC) is the most common subtype of renal cancer. Large-scale metabolomic data have associated metabolic alterations with the pathogenesis and progression of renal carcinoma and have correlated mitochondrial activity with poor survival in a subset of patients. The aim of this study was to determine whether targeting mitochondria-lysosome interaction could be a novel therapeutic approach using patient-derived organoids as avatar for drug response.

**Methods:**

RNAseq data analysis and immunohistochemistry were used to show overexpression of Purinergic receptor 4 (P2XR4) in clear cell carcinomas. Seahorse experiments, immunofluorescence and fluorescence cell sorting were used to demonstrate that P2XR4 regulates mitochondrial activity and the balance of radical oxygen species. Pharmacological inhibitors and genetic silencing promoted lysosomal damage, calcium overload in mitochondria and cell death via both necrosis and apoptosis. Finally, we established patient-derived organoids and murine xenograft models to investigate the antitumor effect of P2XR4 inhibition using imaging drug screening, viability assay and immunohistochemistry.

**Results:**

Our data suggest that oxo-phosphorylation is the main source of tumor-derived ATP in a subset of ccRCC cells expressing P2XR4, which exerts a critical impact on tumor energy metabolism and mitochondrial activity. Prolonged mitochondrial failure induced by pharmacological inhibition or P2XR4 silencing was associated with increased oxygen radical species, changes in mitochondrial permeability (i.e., opening of the transition pore complex, dissipation of membrane potential, and calcium overload). Interestingly, higher mitochondrial activity in patient derived organoids was associated with greater sensitivity to P2XR4 inhibition and tumor reduction in a xenograft model.

**Conclusion:**

Overall, our results suggest that the perturbed balance between lysosomal integrity and mitochondrial activity induced by P2XR4 inhibition may represent a new therapeutic strategy for a subset of patients with renal carcinoma and that individualized organoids may be help to predict drug efficacy.

**Supplementary Information:**

The online version contains supplementary material available at 10.1186/s13046-023-02713-1.

## Background

Deregulation of metabolism is a hallmark of cancer, including clear cell renal cell carcinoma (ccRCC), which is the most common subtype [1–4]. The development of combination therapies with immune checkpoint inhibitors and receptor tyrosine kinase inhibitors (RTKI) has revolutionized the treatment of kidney cancer. However, tumor heterogeneity remains a major challenge [5–7]. Increasing evidence suggests that aberrant lipid accumulation, altered metabolism, and autophagy confer a high-energy state and resistance to ccRCC treatment [3, 8, 9]. Integration of transcriptomic and metabolomic data selected a kidney cancer subgroup independently from histology, characterized by strengthened mitochondrial and weakened angiogenesis-related gene signatures [10]. Metabolomic analysis has identified several metabolites related to the biosynthesis of glutathione, an antioxidant that buffers reactive oxygen species produced by mitochondria, in a particularly aggressive ccRCC subclass [11]. However, the flux and activity of metabolic reactions are fundamental for evaluating the correlation between metabolism and clinical aggressiveness of ccRCC, and only limited data are available [12]. Therefore, targeting mitochondria may represent a novel therapeutic approach to inhibit tumor growth and/or enhance their sensitivity to therapy [13, 14].

The mechanisms leading to the selective disruption of tumor mitochondria are largely unknown. Compared to other intracellular organelles, the dense hydrophobic double-membrane system and negative membrane potential of mitochondria complicate the identification of tumor-selective drug targets [15], similar to the acidic pH of lysosomes. Lysosomes are intracellular Ca^2+^ hubs that are essential for membrane trafficking and signaling [16, 17]. We have previously shown that lysosomal purinergic receptor 4 (P2XR4), an ATP/Ca^2+^ pump [18], plays a key role in energy flux and drives motility and proliferation of endothelial cells [19]. Conversely, blockage of P2XR4 by 5-BDBD inhibits the transport of lysosomal P2XR4 to, and activity on, the cell membrane and reduces tumor angiogenesis [19]. Here, we investigated the role of P2XR4 in metabolic and energy dynamics in ccRCC cell lines. We used patient-derived organoids as personalized drug screening, system and xenograft mouse models to investigate the sensitivity of tumors to P2XR4 inhibition. We reported that P2XR4 inhibition affects tumor cell survival and mitochondrial dynamics in a specific subset of ccRCC characterized by high mitochondrial content and activity, two parameters recently reported to be associated with poor prognosis [11].

## Methods

### Cell culture and sample collection

786-O, A-498, SNC-12, ccRCC cell lines, and HRE, human renal epithelial cells obtained from ATCC (Manassas, VA, USA), were cultured in DMEM medium or RPMI-1640 (Corning, Corning, NY, USA) supplemented with 10% FBS (HyClone, South Logan, UT, USA), 1% penicillin/streptomycin (Gibco, Grand Island, NY, USA), and 2 mM L-glutamine (Gibco, Grand Island, NY, USA) while HREs were growth in Renal Epithelial Cell Growth Medium 2 (Sigma-Aldrich.) Silencing *P2X4,* the oligonucleotides sense:5′GUACUACAGAGACCUGGCUTT3′; antisense:5-′AGCCAGGUCUCUGUAGUACTT3′) were cloned into the BLOCK.it RNAi entry vector together with scramble oligo (from the Invitrogen kit) and transfected into A-498 cells as previously described [20]. Cells transfected for 48 h were selected with G418 at different concentrations according to cell death induction (0.5–100 µg/mL).

### Organoids and samples collection

Patient-derived organoids were generated as previously reported [21] using a protocol approved by the Research Ethics Committee of the University of Campania (2017SZ0064) and was conducted in accordance with the Helsinki Declaration. All patients provided written informed consent prior to acquisition of tissue. Tumor histology was assessed by pathologists. Tissues from kidney carcinomas were minced and subjected to enzymatic digestion in 5 ml of collagenase IV (5 mg/ml) for 1 h at 37 °C. Cell pellets were resuspended in cold organoid culture medium (see Supplementary Information), and 5 × 10^4^ cells in 40 μl droplets with 2% Matrigel were deposited into U 96-well plates. Organoids were passaged every 2–3 weeks at a split ratio of 1:2–1:3 with TripLE (Gibco) and cultured with DMEM/F12, 200 mM GlutaMAx, 1 M Hepes, 1 × B27,100 ng/ml FGF-10 50 ng/ml EGF (PeproTech).

### Image-based drug screening in organoids

For drug screening, 5 × 10^4^ cells in 40 μl droplets with 2% Matrigel were deposited into U 96-well plates and cultured in 100µl of medium. When they reached a size of 200-300 μm after three days of culturing, they were transferred into wells with basket. Test compounds were added to apical and basolateral compartments and equilibrated [22]. Culture medium supplemented with different drug concentrations was replaced every day until harvest time. 20 PDO replicates of individual biopsies were tested with each drug dose. Organoids were exposed to control (0.1% DMSO) or drugs (5-BDBD, everolimus and EZD8055) at different doses for additional 96 h. Images were recorded over time by a Zeiss microscope and sizes of organoids analyzed by ImageJ and CELIGO System (version 1.51). To determine viability, organoids were seeded in 384-well culture plates at 2.5 × 10^4^ cells/15μL/well in organoid medium containing 2% Matrigel. When organoids reached 200µm, 10-fold drug dilution series from 0 to 100 or 200 μM were added to 20 organoid biological replicates. After 96 h, cell viability was quantified using a CellTiter-Glo 3D (ATP) luminescent assay according to the manufacturer’s instructions (Promega), or MTT assay kit and TECAN Infinity 2000 Elisa reader system. The absorbance value of untreated organoid controls (*n* = 20) was considered equal to 1 then the value of organoids treated (*n* = 20 each dose) was reported as ratio of controls. Dose–response curves were fitted using nonlinear squares regression with variable slope (four parameters). Half-maximal inhibitory concentration (IC50) and the fraction of dead cells were determined by the area under the curve (AUC), using GraphPad Prism 9. Statistical evaluation was by two-way ANOVA, with Dunnett’s multiple comparison test comparing each treatment to insensitive PDOs.

### Immunofluorescence

Cyto-spinned organoids (> passage 5) or cells were placed on glass slides, fixed in 4% neutral buffered formalin for 1 h, dehydrated, and permeabilized with 0.5% Triton at 4 °C for 1 min. Slides were blocked in 5% BSA in PBS and incubated with primary antibodies against P2XR4 1:100 PAE-83466 (Invitrogen), CD10 (DAKO Omnis; Clone 56C6), or Ki67 (Dako Omnis; Clone MIB-1) CAIX (H-11 Santa Cruz), and secondary antibodies, both at 4 °C overnight. Nuclei were stained with 4′,6-diamidino-2-phenylindole. Caspase 3 activity was measured using the CaspGLOW™ Fluorescein kit in active caspase-3 (K183-100-N;Nexcelom), and 10 μM Calcein-AM cell-permeant dye (C1430 Thermofisher) was used to stain for cell vitality. Immunofluorescence images were acquired using a confocal Zeiss microscope and analyzed by ZEN2 program. MVs were prepared as previously described [19]. Particle diameters of the EV fractions in the range of 0–1000 nm were analyzed using a Zetasizer Nano ZSP (Malvern Panalytical, Malvern, UK).

### Immunohistochemistry analysis

Consecutive sections of formalin-fixed, paraffin-embeddedtumors were subjected to IHC analysis for the P2XR4 receptor using a DAB substrate kit (Maixin, Fuzhou, China), following the manufacturer’s instructions. The slides were deparaffinized in xylene and rehydrated using graded ethanol to water ratios before staining. All sections were treated with EDTA (pH 8.0) for antigen retrieval and with 3% H_2_O_2_ to inactivate endogenous peroxidases. Sections were incubated with anti-P2XR4 (Invitrogen, PA5-83,466) and CD31 (Dako Omnis; Clone MIB-1) anti-mitochondrial antibodies (SPM198; Abcam) at 1:200 dilution overnight at 4 °C. After washing, sections were stained with a secondary antibody for 30 min at room temperature. Phosphate-buffered saline was substituted for each primary antibody as a negative control. Five random fields were examined under a light microscope. The IHC staining results were independently evaluated by two pathologists who were blinded to the clinical data. The staining intensity was scored as 0 (negative), 1 (low), 2 (medium), and 3 (high). The staining extent was scored as 0 (0% stained), 1 (1%–25% stained), 2 (26%–50% stained), and 3 (51%–100% stained). The final score was determined by multiplying the intensity by the staining extent, and ranged from 0 to 9. Final scores ≤ 4 were considered low staining and > 4 high staining.

### Cell proliferation assay

Cell proliferation was determined by the MTT assay (Sigma, St. Louis, MO, USA), according to the manufacturer’s protocol. Cells (2 × 10^3^) were seeded in 96-well plates and exposed to various concentrations of 5-BDBD for 24 h. Assays were read at 570 nm using a TECAN Infinity 2000 microplate reader (Molecular Devices, Sunyvale, CA). Cell proliferation was measured on different days and reported as absorbance at 570 nm or vitality as ratio of controls.

### Seahorse assay

Mitochondrial and glycolytic rates were measured using the Mitochondrial Stress Test (MST) and Glycolysis Stress Test (GST) Agilent assay kits using a Seahorse XF96 extracellular flux analyzer (Seahorse Biosciences, Billerica, MA, USA). Cells (1.8 × 10^4^ per well) were plated in quadruplicate in XF96 extracellular flux assay plates with 200 μL of XF base medium equilibrated to a pH of 7.4. For organoids assay we follow the Star protocol [22]. Three PDOs with size of 50µm were plated in individual wells of 96 well plates. After adherence for 2 h at 37 °C, media was replaced with GST or MST buffer. For MST, were added the following compounds: oligomycin (1 μM), FCCP (1 μM), and rotenone/antimycin A (0.5 μM). For GST were added: glucose (10 mM), oligomycin (1 μM), and 2-DG (50 mM). Values for each measurement were averaged across quadruplicate wells and reported as the oxygen consumption rate (OCR, pmol O_2_/min) for MST and extracellular acidification rate (ECAR, mpH/min) for GST. Different concentrations of 5-BDBD or 0.1% DMSO were added 5 min before starting MST and GST assays. Data were reported per microgram of protein extracted at the end of the experiment.

### Western blots and immunoprecipitation

Briefly, 30 μg of protein was lysed in ice-cold RIPA buffer with a protease inhibitor cocktail and phosphatase inhibitors (Sigma, MO, USA), loaded on a 12% Tris–glycine SDS polyacrylamide gel, and then transferred to a nitrocellulose membrane. Membranes were blocked with 5% milk in Tris-buffered saline with 0.1% Tween-20 (TBST) for 30 min, and then incubated at 4 °C overnight (1:1000) with primary antibodies: Catalase (PA5-29,183) and LC3BI, II (PA1-16,931) from Thermo Fisher, cleaved CASPASE 9 (20,750), CASPASE 3 (94,530), p62/SQSTM1 (5114), PARP (46D11), GAPDH (D16H11), and β-actin (8H10D10) from Cell Signaling, Ubiquitin (PA5-11,324), and LAMP1 (PA5-95,849) from Thermo Fisher. They were then incubated with goat anti-rabbit/mouse HRP (1:5000, Jackson Immunoresearch, West Grove, PA) for 1 h at room temperature and analyzed using a chemiluminescent kit (Thermo Scientific, Carlsbad, CA, USA). For immunoprecipitation, 50 μg of MVs were resuspended in RIPA buffer and incubated with Protein A/G agarose beads (Santa Cruz sc-2003) conjugated with CD63 antibodies (ab134045, Abcam) for 14 h at 4 °C. After washing and elution, all proteins were loaded onto a 15% SDS polyacrylamide gel.

### MitoTracker, MitoSox and LysoTracker staining

For live imaging, cells (1 × 10^3^ cells) were seeded in 8 μplate IBIDI. 1 μM MitoTracker Red or MitoSox-Red (Invitrogen, Carlsbad, CA) was added to the culture medium and incubated at 37 °C for 30 min. Lysosomal staining was performed using Lyso-Tracker Red DND-99 (Thermo Fisher) at 1 μM for 20 min at 37 °C. 5-BDBD was added at 0.5 μM contemporary to stain dye, using 0.1% DMSO as a control. Stained cells were examined by confocal microscopy (Zeiss LSM 510, Leica, Germany) and photographed simultaneously. For FACS analysis, 1 × 10^5^ cells were treated with 0.1% DMSO or 0.5 μM 5-BDBD for 15 min, 6 h, or 24 h, then stained with 5 μM MitoSox-Red (Thermo Fisher Scientific) for 15 min at 37 °C, and washed in 3% FBS/PBS twice. The fluorescence intensity was measured using a FACSAria III Flow Cytometer (BD Biosciences) [19].

### Real-time qPCR analysis

Total RNA from cultured cells was extracted using TRIzol reagent (Bio-Rad, Hercules, CA, USA) according to the manufacturer's instructions. Two micrograms of total RNA were retrotranscribed (iScript Advanced cDNA Synthesis Kit for RT-qPCR, Bio-Rad) cDNAs were amplified using SYBR Green dye (Green Supermix, Bio-Rad) and with the following primers: Actin (ACTB) as an forward primer 5- ‘CTGGAACGGTGAAGGTGACA3’ and reverse primer 5-AAGGGACTTCCTGTAACAAT. P2XR4 mRNA S- 5’-CATCATCCCCACTATGATCAACA-3′ 714–736 and AS 5-AGCACGGTCGCCATGC-3′ 761–765. The PCR conditions were: denaturation 95 °C 5 s, annealing/extension 60 °C 30 s, melting curve 65–95 °C (0.5 °C increment) for 35 cycles.

### Fura-2-AM calcium flux assay

Live intracellular Ca^2+^ measurement was performed using Fura 2-AM (Invitrogen). Briefly, 1 × 10^4^ A-498 cells were grown in 12 wells plates with DMEM-F12 and 10% bovine serum for 24 h Then washed three times with D-Hanks balanced salt solution without Ca^2+^ (Ca^2+−^free HBSS) or with 2 mM calcium chloride solution and 3 mM ATP (Sigma). Subsequently 2 μM Fura 2-AM with 5 μM 5-BDBD or other ways specify, 0.1% DMSO as control or P2XR7 receptor inhibitor A438079, 30 μM (A9736; Sigma-Aldrich) were added to cultures media for 2 min at 37° C in the dark.. The first 60 s were recorded as basal Ca^2+^ levels. The average fluorescence intensity of each cell in the field (F), normalized to the non-specific background fluorescence (F0), was used to calculate the fluorescence intensity (F/F0). Excitation was performed at 340 nm and 380 nm, and 2-Fura-AM emission was captured at 505 nm. The image fluorescence intensity was measured every 5 s for 2 min after drug treatment and analyzed using a time laps Zeiss microscope with ZEN 2 program. Mitochondrial activity was analyzed using 50 µM carbenoxolone disodium (CBX 3096; TOCRIS). The role of inositol 1,4,5-trisphosphate receptor type 3 (IP3R) was investigated by pretreatment with the 3 μM IP3R inhibitor Xestospongin C (Tocris Bioscience, USA) for 20 min prior to the application of 5-BDBD (5 μM, TOCRIS). The lysosomal contribution to cytosolic Ca^2+^ was tested by pretreatment of cells with an L-L-MA working solution of 10 μM (125,130,250, Thermo Fisher).

### Determination of intracellular oxidative stress

Intracellular oxidative stress after 5-BDBD treatment was determined using a 2′,7′- dichlorofluorescein-diacetate (DCFDA/ H2DCFDA- Cellular ROS Assay Kit (ab113851, Abcam). The cells were seeded in 96-well plates at (1 × 10^3^) cells/well and cultured overnight. Cells were then preincubated with 50 μM DCFDA for 15 min and treated for an additional 15 min with 0.5 μM 5-BDBD, or 0.1% DMSO a negative control, or 100 μM H_2_O_2_, or 25 mM CaCl_2_, or carbonyl cyanide m-chlorophenyl hydrazone 10 μM CCCP as positive controls [23]. Fluorescence images were captured by Zeiss confocal microscope and analyzed the intensity with ZEN 2.0 program (Germany). The mean of fluorescence intensity at 488 nm was determined in triplicate well experiments using TECAN Infinity 2000 Elisa reader. Glutathione determination was performed with glutathione colorimetric detection kit (Invitrogen) following kit guide accordingly with to manufacture’s instruction.

### Mitochondrial Ca^2+^ measurement

Mitochondrial Ca^2+^ levels were measured using the Rhod-2 AM dye (Abcam). For flow cytometry, 10^5^ plated cells were culture in 0.1% DMSO or 0.5 μM 5-BDBD (treated cells) with Rhod-2 AM dye (5 μM) for 30 min at 37 °C. Cells were then washed twice, suspended in 500 μL of PBS, and analyzed by flow cytometry. For live microscopy image, cells were grown in microplates Ibidi. Then 5 μM of Rhod-2 AM dye and 1 μM and MitoTracker green were added to the media (Cell Signaling Technologies) for 10 min, followed by 0.1% DMSO or 5 μM 5-BDBD for an additional 20 min. The images were captured using a confocal Zeiss microscope and analyzed by ZEN 2.0 program (Germany).

### Mitochondrial membrane potential

Control and cells treated with 5-BDBD (0.5 μM for 15 min) were resuspended in 1 mL medium containing 5 μM JC-1 mitochondrial membrane potential probe (e-CK-A301 Elabscience). After incubation at 37° C for 30 min, the cells were washed twice, resuspended in 500 μL of PBS, and analyzed by flow cytometry. The percentage of cells that exhibited red and green fluorescence was quantified. Carbonyl cyanide m-chlorophenyl hydrazone (CCCP), an inhibitor of mitochondrial oxidative phosphorylation that causes ROS-induced mitochondrial depolarization [23], was used as a positive control. The cells were treated with 10 μM CCCP for 15 min prior to flow cytometry. Fixed cells were photographed using a confocal microscope (Zeiss, Germany) and analyzed using ImageJ.

### Apoptosis assay

Cells (1 × 10^5^) were treated with 0.1% DMSO or 5 μM 5-BDBD for 24 h, dissociated, washed in PBS, and incubated in Annexin V binding buffer containing annexin V-FITC and PI (BD Biosciences) for 15 min at room temperature. The stained cells were diluted 1:5 in binding buffer and analyzed using a FACSAria III Flow Cytometer (BD Biosciences).

### In vivo mouse model

A tumor xenograft model was established as described in our previous report [19]. Briefly, A-498 cells (5 × 10^6^ cells per mouse) were subcutaneously injected into the right flanks of 6-week-old BALB/c female nude mice. Equal-sized groups were generated using a randomized experimental design. Mice (*n* = 3 per cage) were housed in a temperature-controlled (22–24 ℃) environment with a relative humidity of 60–70% and a 12 h light/12 h dark cycle. All animals received water and food ad libitum. When the tumor size reached approximately 100 mm^3^, mice were randomly assigned to treatment groups (PBS or 10 mg/kg 5-BDBD administered by intraperitoneal injection every 3 days for 55 days, *n* = 5 per group). Before each administration, the body weights of the mice were monitored. Simultaneously, the tumor volumes were measured and calculated according to the formula: tumor volume (mm^3^) = 0.5 × (length of tumor) × (width of tumor). At the end of the experiment, the mice were killed by an overdose of pentobarbitone (200 mg/kg), and the tumors were dissected, fixed, and used for immunohistochemical analysis.

### Statistical analysis

All data were analyzed by one-way ANOVA with Tukey’s post hoc analysis using or Student’s t test using GraphPad Prism 9 (LA USA). Dose–response curves were fitted using nonlinear squares regression with variable slope (four parameters). Half-maximal inhibitory concentration (IC50) and the fraction of dead cells were determined by the area under the curve (AUC), using GraphPad Prism 9. Statistical evaluation was by two-way ANOVA, and Dunnett’s multiple comparison test was used to compare each treatment to control. results are expressed as mean ± SEM or SD. The threshold (α) of significance was set at *p* < 0.05.

## Results

### P2XR4 overexpression in ccRCC correlates with poor prognosis.

**Fig. 1:**
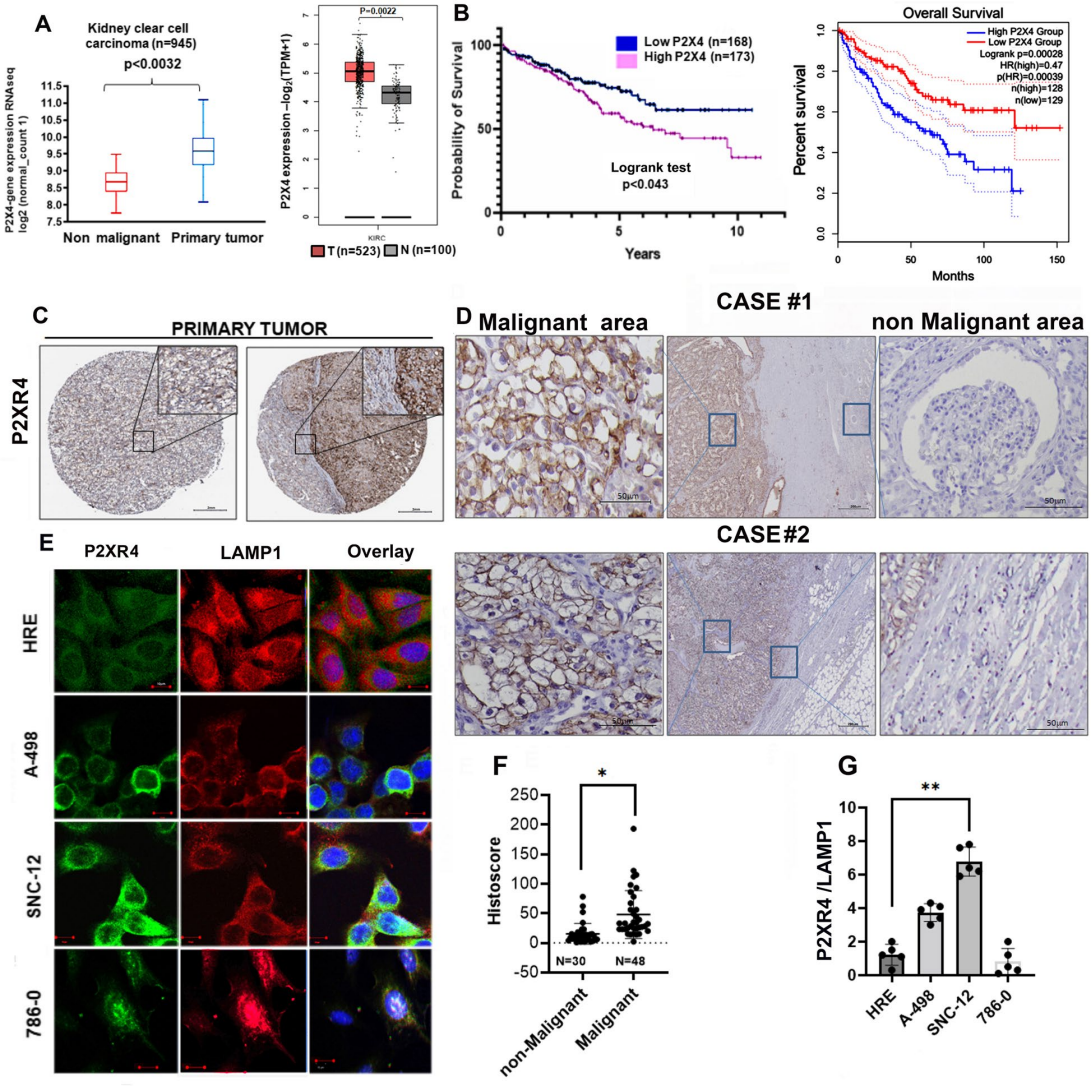
P2X4 gene and protein overexpression in clear cell renal carcinoma correlates with poor patient prognosis. **A** Left: *P2X4* RNAseq analysis in clear cells renal carcinoma primary tumors (KIRC) cohort from the Cancer Genome ATLAS (TCGA) *n* = 945 with Xena browser, compared to adjacent non-tumoral tissues. Right: *P2X4* RNAseq in KIRC cohort from the Cancer Genome ATLAS, (*n* = 523) analyzed with GEPIA browser compared to non-tumoral tissues (*n* = 100). **B** Left**:** Kaplan–Meier estimates overall survival in KIRC patient cohort (grade I-III) with high and low *P2X4* mRNA expression (logrank test *p* < 0.043). Right**:** Kaplan–Meier estimates overall survival among a cohort of 258 tumor grade (I-III) expressing high and low *P2X4* mRNA level (logrank test, *p* < 0.00028). **C** Expression of P2XR4 protein assessed by immunohistochemistry in the human protein ATLAS database. Right: Representative image of clear cells renal carcinoma grade I showing moderate staining for P2XR4 antibody. Left: Strongly stained clear cell renal carcinoma grade I; Scale bar = 200 μm, insert is higher magnification, scale bar = 50 μm. **D** Representative images showing P2XR4 staining of clear cell renal carcinoma tissues stage III from two of our clinical cases. Middle: low magnification image (Scale bars = 200 µm) showing areas of tumor and non-malignant tissues; on both sides the higher magnifications (scale bars = 50 µm). **E** Immunofluorescence of P2XR4 (green), LAMP1 (red), and nuclei DAPI (blue) in normal renal epithelial cells (HRE) and clear cell carcinoma cell lines (A-498, SNC-12, and 786–0) (Scale bars = 20 μm). **F** Histoscores scatterplot for P2XR4 protein in non-malignant (*n* = 30) and malignant KIRC patients’ (*n* = 48 using QuPath). Statistical significance was determined by unpaired two-tailed *t*-test; *p ≤ 0.05. **G**, Bar graph showing relative immunofluorescence intensity of P2XR4 protein in individual cell lines stained with fluorescence-labeled antibodies. At least 20 cells for each cell lines by unpaired two-tailed *t*-test; **p ≤ 0.01

Given that our previous study had established that P2XR4 affects endothelial mitochondrial activity [19], we now investigated its role in ccRCC metabolism. We began by evaluating *P2XR4* gene and protein expression in human ccRCC. Bioinformatic analysis of the RNAseq database of human samples from The Cancer Genome Atlas (TCGA, accessed using the Xena browser), indicated that *P2X4* mRNA was upregulated in a cohort of primary ccRCC (*n* = 945), compared to adjacent non-tumor tissues (Fig. 1A, p = 0.0032). This was also confirmed using the GEPIA browser (Fig. 1A, p = 0.0022). P2X4 mRNA was a variable independent of tumor grade (I-III) and its high expression correlated with poor overall survival (OS) (*p* = 0.043 and = 0.00028) (Fig. 1B). P2XR4 protein, assessed by immunohistochemistry in the Human Protein Atlas database (ATLAS) showed positive staining in clear cell carcinoma sections (Fig. 1C). The signal was moderate in 49% and strong in 19% of the cases, and predominantly localized in the cytoplasm and on membranes. Immunohistochemistry of our primary ccRCC tissues confirmed greater P2XR4 expression in tumors than in adjacent nonmalignant tissues, as shown in representative images (Fig. 1D). Additionally. microarrays featuring normal (*N* = 30) and malignant tissues (*N* = 48) confirmed a significantly enhanced expression of P2XR4 protein reported as a histoscore in tumor compared to normal tissues (Fig. 1F). In A-498 and SNC-12 cells, P2XR4 colocalized with the main lysosomal protein, LAMP1, whereas it was expressed at lower levels in the 786–0 clear carcinoma cell line and in normal epithelial renal cells (HRE) then in A-498 and SNC-12 cells (Fig. 1E, G).

### Mitochondrial metabolism in ccRCC

**Fig. 2:**
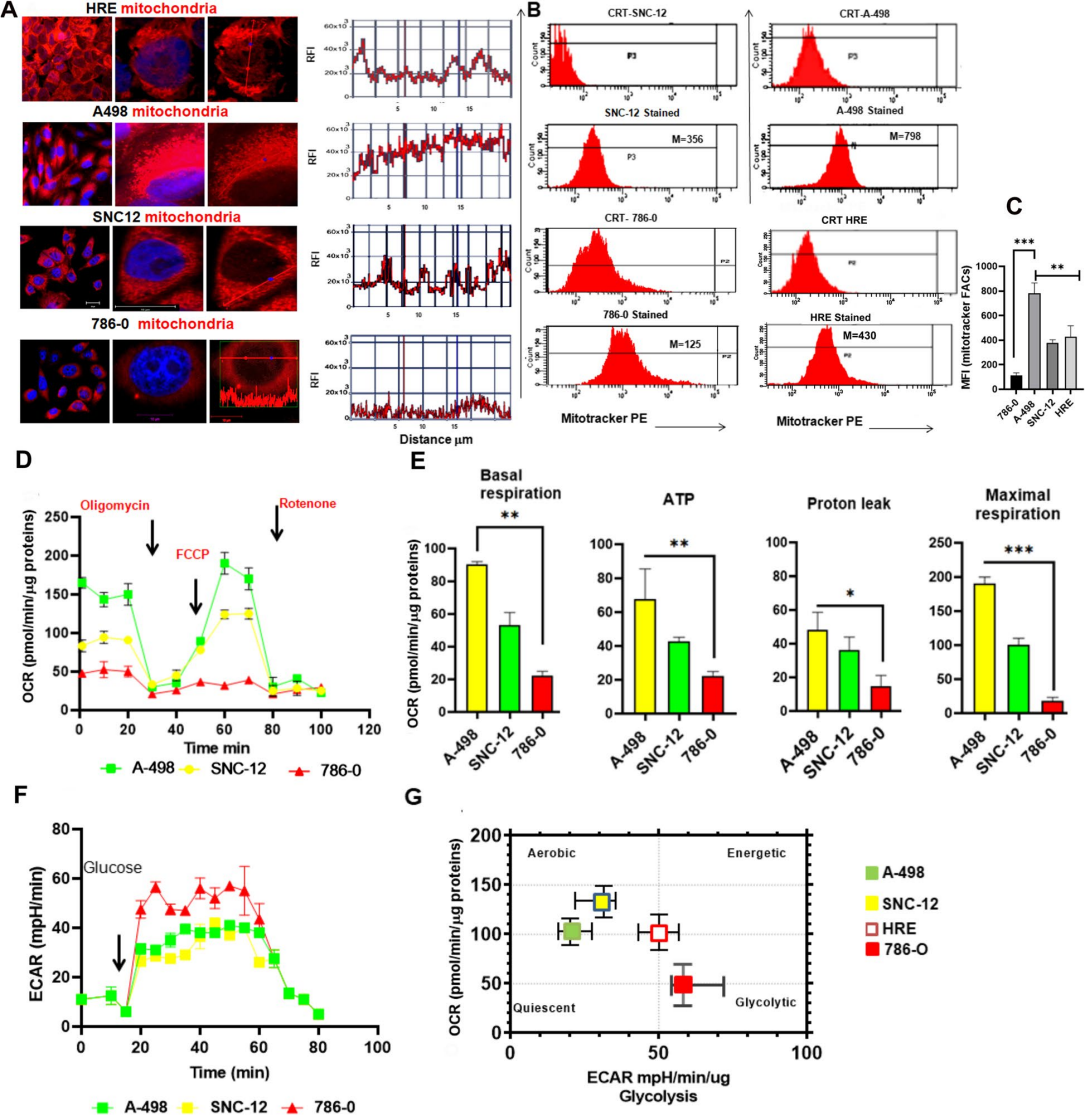
Mitochondrial mass and activity in tumor cells. **A** Left: MitoTracker Red staining of normal human renal epithelial cells (HRE) or A-498, SNC-12 and 786–0 clear cell carcinoma cells (Scale bars = 10 μm). Right: Relative fluorescence intensity (RFI) of MitoTracker in single cell along the diameter. **B** Representative FACS profile of mitochondrial mass labeled with MitoTracker in SNC-12, 786–0 and A-498 cells, reported as median of fluorescence intensity (MFI). CRT indicates the unstained control of each cell line. **C** Graphical representation of mean fluorescence intensity (MFI) measured by FACS. **D** Oxygen consuming rate (OCR) by mitochondrial stress test (MST using Seahorse system) in A-498, SNC-12 and 786–0 cells plated in 12 × multiwell plates at 1 × 10.^5^ cells/well. Data are reported per µg of protein under basal conditions and in response to mitochondrial inhibitors (oligomycin; FCCP; rotenone). **E** Quantification of basal respiration, ATP production, proton leak and maximal respiration in tumor cells. Basal respiration is the value just before oligomycin injection, minimal respiration is the lowest value after oligomycin injection, and maximal respiration is the highest value after FCCP injection. All values were calculated after subtraction of non-mitochondrial respiration. **F** Extracellular acidification rate (ECAR) reported as milli pH per minute (mpH/min) under basal conditions in A-498, SNC-12 and 786–0 cells over the time. **G** ECAR in milli pH per minute (mpH/min)/ µg under basal conditions over the time vs. OCR under conditions of basal respiration in A-498, SNC-12, 786–0 and HRE cells. Abbreviations: Oligo, oligomycin; FCCP, carbonyl cyanide-p-trifluoromethoxyphenyl hydrazone; Rotenone. Data are mean ± SEM. of three independent experiments in triplicated. *P*-values were calculated by Mann–Whitney U or Student's t-tests. **p < 0.01

Correlative analysis showed a significant association between *P2X4* gene expression and glutathione peroxidase (*GPX4*, *p* = 6.8^−6^), superoxide dismutase (*SOD2*, *p* = 2.3^–9^) involved in the detoxification of radical oxygen, and exokinase 2 (*HK2*, *p* = 7^−12^), which regulates the integrity of mitochondrial membrane (Suppl. Figure 1). We therefore investigated the role of P2XR4 in intracellular Ca^+2^ homeostasis and mitochondrial activity in ccRCC cells [24]. Confocal microscopy and FACS analysis using mitotracker staining showed twofold greater mitochondrial fluorescence intensity in A-498 cells than in SNC-12 cells, with both being higher than that in normal renal epithelial cells (HRE) (Fig. 2A,B). The lowest mitochondrial fluorescence was observed in 786–0 cells (Fig. 2A,B) consistent with the mean fluorescence intensity measured by FACS (Fig. 2B,C). Then mitochondrial activity was determined by dosing the oxygen consumption rate (OCR) over time and respiratory parameters calculated using inhibitors of mitochondrial function (oligomycin, FCCP and Rotenone + Antimycin) (Fig. 2D). Results showed that A-498 and SNC-12 cells had higher OCRs than 786–0 cells (Fig. 2D). OCRs associated with maximal respiration of SNC-12 and 786-O cells were 2.3- and fivefold smaller than those of A-498 cells, respectively, as well as ATP production (Fig. 2E). In contrast, the extracellular acidification rate (ECAR) in 786–0 cells was 1.8-and 2.0-fold greater than that in A-498 and SNC-12 cells, respectively (Fig. 2F). More importantly, in A-498 and SNC-12 cell lines, the OXOPHO rate (monitored as OCR) was greater than glycolysis activity measured as extracellular acidification rate (ECAR), indicating that in these cells, mitochondrial metabolism is the principal energy source instead of glycolysis (Fig. 2G). In normal HRE cells, OCRs and ECAR were well-balanced, whereas in 786–0 cells glycolysis (ECAR) was the principal source of energy production (Fig. 2G).

### P2XR4 roles in mitochondrial calcium homeostasis

**Fig. 3:**
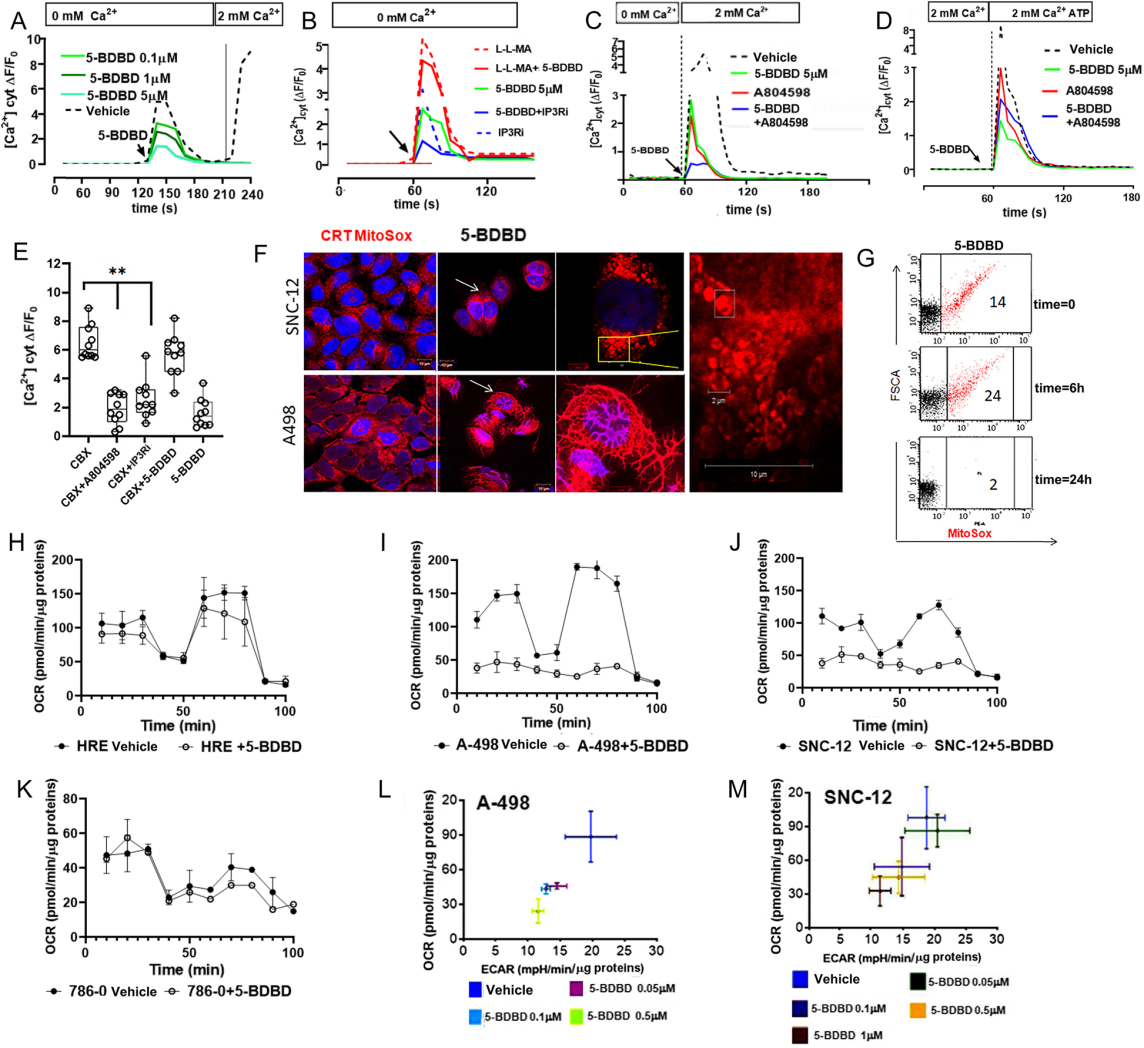
P2XR4 receptor regulates intracellular calcium. Cytosol Ca^2+^ dosage in live A-498 cells traced by Fura-2 AM over the time. **A** Baseline reading in Ca^2+^ free conditions were established for 2 min, then cells were recorded for additional 2 min without or with P2XR4 inhibitor (5-BDBD at different concentration green lines). **B** Intracellular calcium response to 5-BDBD, or inositol 3 phosphate receptor inhibitor (IP3Ri), or lysomotropic inhibitor (L-L-MA) of A-498 cells incubated in Ca^2+^-free medium. **C** Intracellular calcium levels in cells stimulated with 2 mM Ca^2+^-containing medium in presence of either a P2XR7 inhibitor (A804598) or 5-BDBD or combination of both. **D** Intracellular calcium levels in cells stimulated with 3 mM ATP and 2 mM Ca^2+^-containing medium in presence of either a P2XR7 inhibitor (A804598) or 5-BDBD or both. **E** Box plot of intracellular calcium in A-498 cells following inhibition of mitochondria (CBX), P2XR7 (A804598), P2XR4 (5-BDBD), or IP3Ri, as indicated. Data are reported as ratio between F and F0 (340/380 nm fluorescence of Fura 2-AM) recorded in controls, *n* = 51 cells, A804598, *n* = 50 cells, 5-BDBD *n* = 39 cells and IP3Ri inhibitor *n* = 45 during the 2 min of stimulation**.** *P*-values were calculated by Mann–Whitney U **p < 0.01. **F** Pharmacological blockage of P2XR4 results in mitochondrial morphology change. Representative confocal image of SNC-12 and A498 cells mitochondria stained with MitoSox (red) and DAPI for nuclei (blue). After treatment with 5-BDBD for 10 min the mitochondria appeared fused in filaments in A-498 cells or with large cristae in SNC-12 cells. Scale bars = 10 µm. **G** Representative flow cytometry analysis of mitochondria stained with MitoSox (red) from A-498 cells after different time of 5 µM 5-BDBD treatment, as indicated. The percentage of positive cells is reported in each quadrant. **H** Oxygen consumption rate (OCR) with the Mito Stress Test kit in HRE cells incubated with or without 5 µM 5-BDBD over the time. **I** and **J** OCR measurements in A-498 and SNC-12 cells with or without 5 µM 5-BDBD. **K** OCR in 786–0 cells with or without 5 µM 5-BDBD. **L** and **M** OCR vs extracellular acidification rate (ECAR) in A498 and SNC-12 cells treated with 5-BDBD at different concentrations, as indicated. Data represent mean ± SD of three independent experiments performed in triplicate

Since intracellular Ca^2+^ is necessary for mitochondrial activity, we focused on the contribution of P2XR4 to cytosolic calcium [Ca^2+^]_cyt_ homeostasis. For this purpose, we measured [Ca^2+^]_cyt_ in A-498 cells, which showed the greatest mitochondrial activity among the cancer cell lines tested, using Fura-2 AM as an indicator. Cells were grown in Ca^2+^-free media to stimulate Ca^2+^ release from storage and treated for 2 min with different doses of 5-BDBD, an inhibitor of P2XR4. 5-BDBD treatment reduced the cytosol calcium concentration compared to that in the controls in a dose-dependent manner (Fig. 3A). In order to understand whether 5-BDBD treatment blockade the calcium release from lysosomes or endoplasmic reticulum (ER) we grew cells in Ca^2+^ free and blocked its release from organelles with selected inhibitors (Fig. 3B). 5-BDBD treatment following the IP3R antagonist reduced [Ca^2+^]_cyt_ peak indicated that 5-BDBD did not modulate ER Ca^2+^ storage. In contrast the addition of 5-BDBD- did not reduce [Ca^2+^]_cyt_ after lysosomal damage induced by L-leucyl-L-leucine methyl ester (L-L-MA), a lysosomotropic agent (Fig. 3B). These data indicated that P2XR4 regulates the flux of lysosomal Ca^2+^into the cytoplasm. In subsequent experiments, Ca^2+^ uptake by cells was stimulated by adding 2 mM Ca^2+^ to the medium by itself or in combination with 3 mM ATP (Fig. 3C, D). Again, treatment with 5-BDBD decreased Ca^2+^ in the cytosol (Fig. 3C, D). Simultaneous administration of 5-BDBD and A084598, a P2XR7 inhibitor, strongly reduced [Ca^2+^]_cyt_ compared to 5-BDBD alone, when ATP was absent, indicating their synergy (Fig. 3C). Additionally_,_ blocking mitochondrial activity by CBX enhanced intracellular Ca^2+^ via P2XR7 and endoplasmic reticulum receptor; however, this was independent of P2XR4 (Fig. 3E). Indeed, intracellular Ca^2+^ remained unchanged when 5-BDBD was added together with CBX, compared to 5-BDBD alone (Fig. 3E). These data indicated that the contribution of P2XR4 to intracellular Ca^2+^ homeostasis is linked to mitochondrial activity. Moreover, at an early time point (10 min), 5-BDBD treatment altered the mitochondrial shape (Fig. 3F). In contrast to the random distribution in the cytoplasm, mitochondria appeared fused in long filaments as shown by MitoSOX-Red staining (Fig. 3F) and after 24 h the mitochondrial mass was reduced (Fig. 3G) [25]. Accordingly, P2XR4 inhibition reduced basal and maximum mitochondrial oxygen consumption rates in A-498 and SNC-12 cells compared to HRE and 786–0 cells (Fig. 3H-K). The OCR reduction occurred in concentration- dependent manners without increase of glycolysis (ECAR) (Fig. 3L, M). We conclude that P2XR4 contributes to the [Ca^2+^]_cyt_ homeostasis required to maintain mitochondrial cellular respiration.

### P2XR4 promotes metabolic stress resistance

**Fig. 4:**
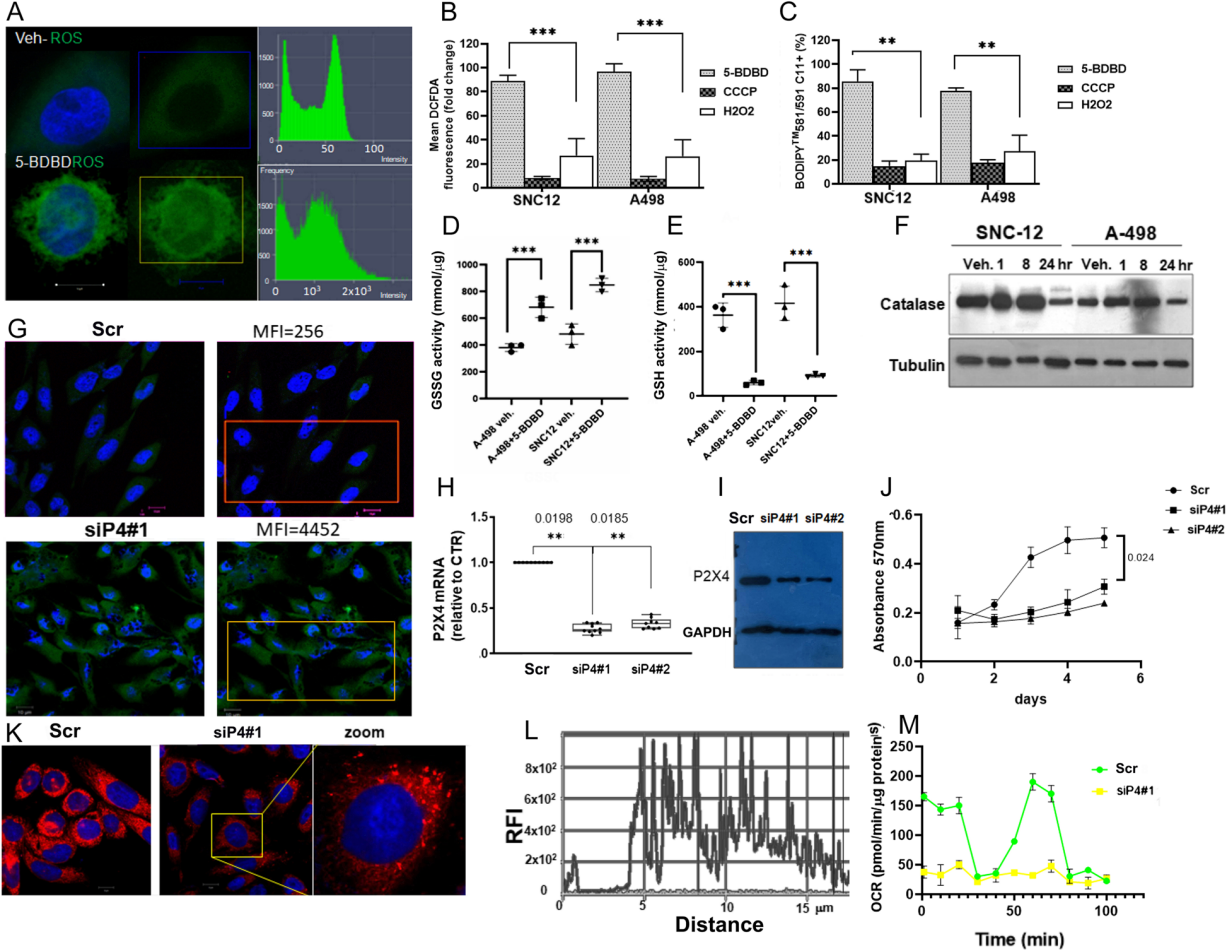
P2XR4 activity protects from ROS. **A** Left: Representative confocal image showing ROS production (DCFDA fluorescence) in a single cell assay. TOP A-498 cell control treated with vehicle (DMSO) or exposed for 15 min to 5-BDBD (bottom). Right: cells stained with DCFHDA green were boxed and fluorescence quantified were Graphical represented as relative fluorescence intensity of ROS in the square area. **B** Graphical representation of mean DCFDA fluorescence intensity (recorded as λ_Ex/Em_ = 495/529 nm) reported as fold change over control vehicle in SNC-12 and A-498 cells, treated for 10 min with 0.5 μM 5-BDBD or positive control (500 μM H_2_O_2_) or negative controls (10 μM CCCP, protonophore *m*-chlorophenylhydrazone). **C** C11-BODIPY^581/591^ was used to index lipid peroxidation. SNC-12 or A-498 cells were incubated with 2.5 µM C11-BODIPY.^581/591^ for 15 min after exposure to 5-BDBD (0.5 μM), H_2_O_2_ (500 μM), and negative control 10 μM CCCP (protonophore *m*-chlorophenylhydrazone) for 10 min. Data are reported as fold change to vehicle treated cells. **D**, **E** GSSG and GSH colorimetric assay in A-498 and SNC-12 cells treated for 15 min with vehicle or with 5-BDBD. Data are reported as millimol of GSH or GSSG / µg of protein extracts. **F** Western blot of protein extracts from A-498 and SNC-12 cells collected at different time points from 5-BDBD treatment stained with Catalase antibody. Tubulin was used as loading control. **G** Representative confocal images showing ROS production (DCFDA fluorescence) in scramble transfected control A-498 cells (Scr) or in silenced *P2X4* clone (siP4#1). The mean relative fluorescence intensity measured (MFI) in the squared area is indicated. **H** qRT-PCR dosage of *P2X4* mRNA in scramble control A-498 cells (Scr) and in two different silenced clones, siP4#1 and siP4#2. **I** Western Blot analysis of proteins from scramble control A-498 cells (Scr) and in two silenced clones siP4#1 and siP4#2 with P2XR4 antibodies and GAPDH as loading control. **J** Cell proliferation assay in A-498 scramble transfected cells (Scr), and in siPX4 clones 1 and 2 assessed by MTT at different days of cultures (*n* = 3) reported as OD at 570 nm. **K** MitoTracker Red staining for mitochondria in scramble transfected A-498 cells (Scr) and siP4#1 clone (scale bar = 10 μm). **L** Relative fluorescence intensity for MitoTracker Red along the diameter of a siP4#1 single cell. **M** Oxygen consumption rate (OCR) measurements in scramble transfected A-498, (Scr) and in siP4#1 clone with the Mito Stress Test. Data B, C, D, E, H and J M represent as mean ± SD of three independent experiments performed in triplicate. Student’s t-test ** *p* < 0.01.***p < 0.001

Reactive oxygen species (ROS) damage several cellular functions. Mitochondria play a role in the detoxification of ROS through specific enzymes, such as manganese superoxide dismutase (MnSOD/SOD2) and Catase [26]. Therefore, we investigated whether P2XR4 inhibition affects these pathways. First, we evaluated whether pharmacological inhibition of P2XR4 affected ROS production. A few minutes after 5-BDBD treatment, a significant increase in ROS was measured by DCFDA fluorescence in A-498 and SNC-12 cells (*p* < 0.001) (Fig. 4A, B) and lipid peroxidation (*p* < 0.01 (Fig. 4C). ROS levels were higher after 5-BDBD treatment than in response to oxidative stress induced by H_2_O_2_ (Fig. 4B). Consistent with this observation, 5-BDBD treatment significantly increased the oxidant form of glutathione (measured as GSSG activity) (*p* < 0.001), an important regulator of redox homeostasis, and reduced the levels of detoxifying enzymes, such as Catalase, compared to control cells (Fig. 4D-F) [27]. No significant effects on SOD protein expression were observed (*data not shown*). The silencing of P2XR4 recapitulated most of the above findings compared to scramble clone, such as the accumulation of radical oxygen species, reduction of growth, and reduction of the mitochondrial oxygen consumption rate (Fig. 4G-M).

### P2XR4 inhibition induces mitochondrial pore dysfunction

**Fig. 5:**
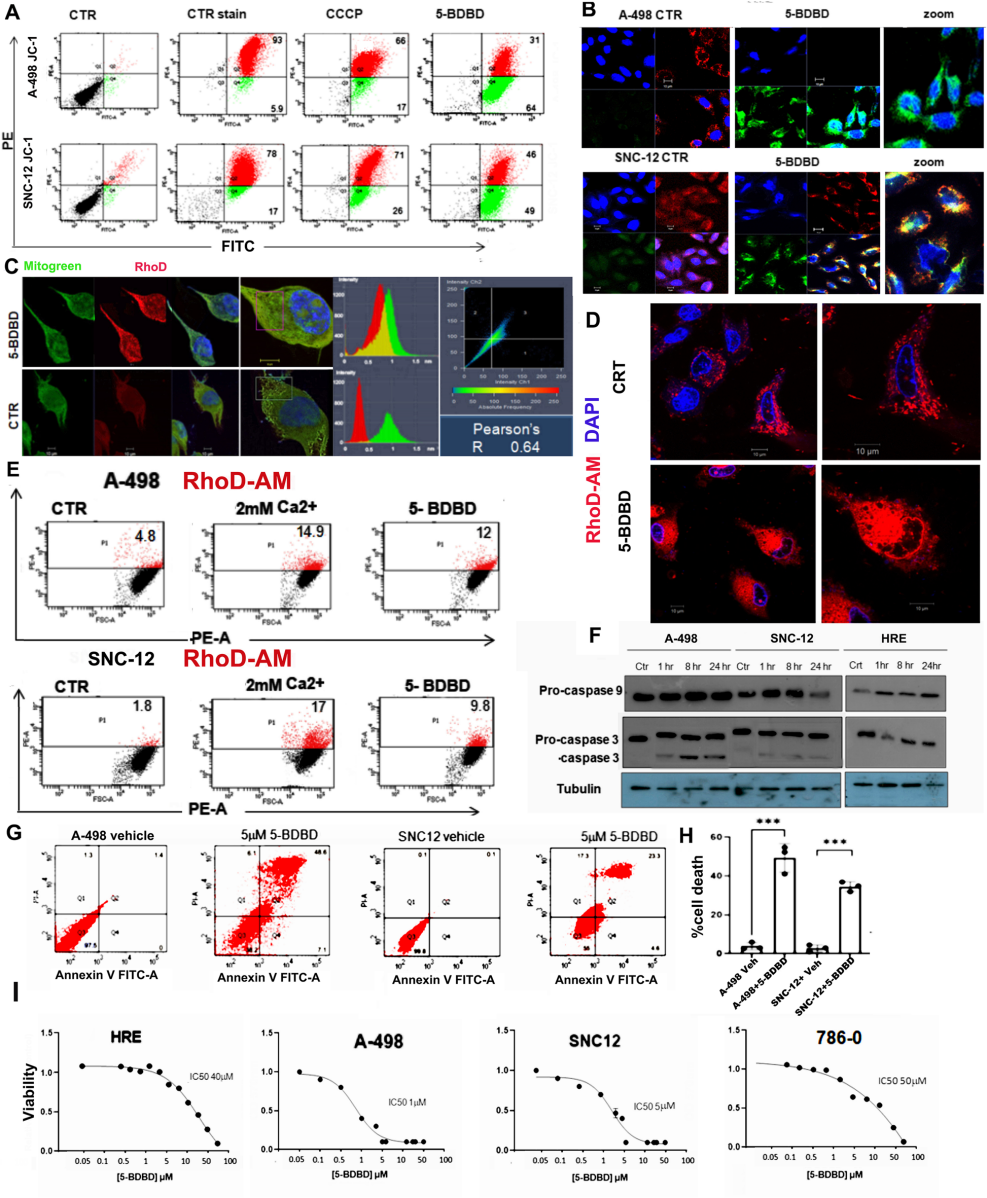
P2XR4 protects mitochondrial membrane from oxidation and calcium overload. **A** Determination of mitochondrial membrane potential (ΔΨm) in A-498 (top) and SNC-12 cells (bottom) with JC-1 staining, using fluorescence cell sorting. The cells were treated for 15 min with CCCP (positive control), 5-BDBD, or vehicle. The high mitochondrial membrane potential (Δ*Ψ*_m_), in red corresponds to dimers of JC-1, and the low ΔΨm in green corresponds to the JC-1 monomer. Percentages of red and green potentials (ΔΨm) are indicated in each quadrant. **B** Representative images of A-498 and SNC-12 cells incubated with or without 5-BDBD for 15 min and stained with JC-1. Red staining indicates high mitochondrial membrane potential and green staining indicates low potential (ΔΨm). Scale bar = 10 µm. **C** Representative image of a single cell assay of A-498 cells treated with 5-BDBD for 15 min (upper panel) or vehicle- (lower panel) stained with MitoTracker green and Rhodamine-AM red as indicators of mitochondrial calcium accumulation. Correlation between green and red fluorescence intensity in single cell assay with Pearson test; *R* = 0.64 (scale bar 10 µm). **D** Representative confocal image of Rhodamine-AM (red) stained mitochondrial calcium in A-498 cells treated for 15 min with vehicle (control) or 5-BDBD (scale bar 10 µm). **E** Flow cytometry quantification of mitochondrial calcium accumulation by Rhodamine-2AM (red) in vehicle control, A-498, and SNC-12 cells, or cells exposed to 2 mM Ca^2+^ with or without 5-BDBD. The percentage of Rhodamine-2 AM positive mitochondria (red) is indicated in the corresponding quadrant. **F** Western blot analysis of pro-Caspase 9 and 3 in protein extracts from A-498, SNC-12, and control HRE cells treated with 5-BDBD for different times. **G** Early and late apoptosis in A-498 and SNC-12 cells treated with 5-BDBD or vehicle for 24 h stained with FICT-Annexin V and PI and analyzed by flow cytometry. **H** Quantification of early and late apoptotic cells. Data are mean ± SD. of three independent experiments performed in triplicates ***p < 0.001. Statistical analysis was performed using one-way ANOVA followed by Turkey’s post-hoc test. **I** Dose response curve and IC50 for 5-BDBD determined by MTT assay reported as vitality / control, A-498, SNC-12, HRE; and 786–0 cells. Data are presented as the mean ± SD of three independent experiments performed in triplicates

To verify whether mitochondrial dysfunction induced by selective P2XR4 inhibition was associated with alterations in membrane potential, we used the potential-sensitive JC-1 dye (Fig. 5). Flow cytometry showed a significantly increased ratio between depolarization (green) and oxidation (red) in treated cells, compared to cells maintained in 2 mM Ca^2+^ or treated with CCCP, a compound known to alter the membrane potential (Fig. 5A). Similarly, confocal images of cells showed a decrease in mitochondrial membrane oxidant potential (red) after 5-BDBD treatment and increased depolarization of membranes (green) (Fig. 5B). As mitochondrial potential is essential for regular Ca^2+^ uptake, we hypothesized that 5-BDBD treatment could affect uncontrolled mitochondrial Ca^2+^ entry. Using Rhodamine 2-AM as a sensor of mitochondrial Ca^2+^, we detected an overload of mitochondrial Ca^2+^ after 15 min of 5-BDBD treatment dosed by fluorescence and FACS (Fig. 5C-E) which ultimately resulted in activation of mitochondrial Caspase 3 (Fig. 5F). Apoptotic FACS screening by annexin V-FITC and PI staining of SNC-12 and A-498 cells after 5-BDBD treatment, resulted in 44% and 52% of cell death, respectively (Fig. 5G,H). Moreover, the viability of cancer cell lines with high mitochondrial activity (such as A-498 and SNC-12) was sensitive to 5-BDBD treatment in a concentration-dependent manner, with an IC_50_ of 1 µM and 5 µM after 24 h, respectively (Fig. 5I). In contrast, normal epithelial cells and 786–0 cells with lower mitochondrial activity were less sensitive (IC_50_ = 40 and 50 µM) (Fig. 5I). These results indicate that the efficacy of 5-BDBD is depending on the molecular characteristics of the cancer cells and P2XR4 protects against ROS via a mitochondrial pathway.

### P2XR4 contributes to lysosomes integrity and function

**Fig. 6:**
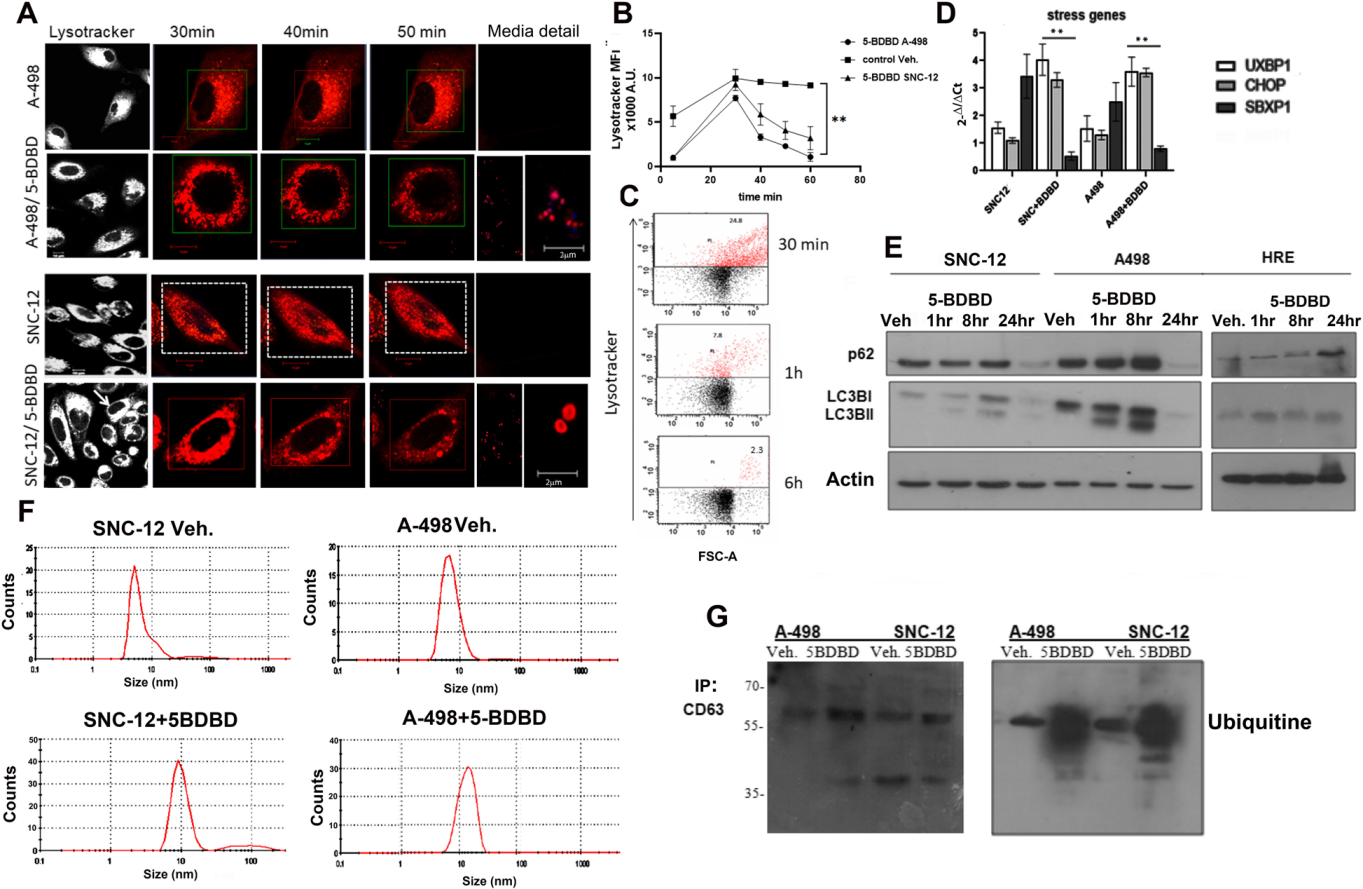
Blocking P2XR4 activity induces lysosomal membrane damage. **A** Time-laps confocal microscopy live images of A-498 and SNC-12 cells stained for lysosomes with lysotracker (red). Images were taken after different time of exposure to 5-BDBD, as indicated. Scale bars = 10 µm. **B** Graphical representation of mean ± SE of lysotracker (red) fluorescent intensity of *n* = 25–30 cells per sample over time in three independent experiments ± SD ** *p* < 0.01. **C** Flow cytometry quantification of lysotracker stained A-498 cells after different time of 5-BDBD treatment*.* **D** RT/PCR quantification of mRNAs of stress genes CHOP, UBXP1, and SPBX1 in SNC-12 and A-498 cells incubated without or with 5-BDBD for 1 h. Data are reported with 2-delta/delta ct method ± SD of three independent experiments; ** *p* < 0.01. **E** Representative Western blot of protein extracts from SNC-12, A-498 and HRE cells treated or not with 5-BDBD for different times and analyzed with LC3BI/II and p62 antibodies, as indicated. Western blot bands were normalized with actin. **F** Zetasizer nano analysis of nanobody selected with CD63 antibody from SNC-12 and A-498 cell culture media after treatment with vehicle or 5-BDBD. **G** Western blot of extracellular CD63 + nanobody prepared from cultured media of A-498 and SNC-12 cells treated with 5-BDBD and control vehicle (0.1% DMSO) immunoprecipitated with CD63 antibody (left) and Western blot analyzed with poli-Ub antibody (right)

Lysosomes are acidic, membrane-bound organelles containing hydrolytic enzymes and stored Ca^+2^ [28]. As in resting cells P2XR4 is mainly localized in lysosomes, we investigated whether mitochondrial Ca^2+^ overload in renal cancer cells is also linked to lysosomal damage induced by 5-BDBD. We evaluated lysosome integrity before and after prolonged treatment with 5-BDBD using lysotracker staining, as this dye normally translocates rapidly to the inner membrane of acid organelles (Fig. 6A). The A-498 and SNC-12 cells were strongly stained by lysotracker, indicating a high number of acidic organelles. A time course experiment in living cells treated with 5-BDBD showed a peak of acid vesicles at 30 min, then a reduction in lysosome staining (Fig. 6A, B), coinciding with an extracellular accumulation of acid vesicles (see staining of medium in Fig. 6A) [29]. Lysotracker FACS analysis confirmed an increase in acid vesicles in treated cells at early time point, whereas after 6 h reduced (Fig. 6C). RT-PCRs confirmed the accumulation of stress gene mRNAs (Fig. 6D) and stable expression of autophagy proteins, such as p62 and the accumulation of both LC3BI and its active isoform, LC3BII, after several hours of 5-BDBD treatment (Fig. 6E). The observation that LCB3II proteins did not increase and p62 was stable for 8 h suggested that autophagy was not responsible for the loss of lysosomal integrity. Because the molecular mechanism to eliminate damaged organelles is mediated by an ubiquitin (Ub)-dependent pathway [29, 30], we determined whether this event occurred also during treatment with 5-BDBD. For this purpose, culture media were immunoprecipitated for CD63 antigen and analyzed (Fig. 6F, G). Nanosize measurements indicated that CD63 ^+^ nanobody from 5-BDBD treated cells were bigger in size than controls whereas Western blotting indicated that they were polyubiquitinated, compared to untreated controls (Fig. 6F, G). Collectively, these results indicate that 5-BDBD affect the autophagy mechanism and releasing polyubiquitinated small membranes.

### Therapeutic efficacy of P2XR4 inhibition in patient-derived organoids is related to mitochondrial activity

**Fig. 7:**
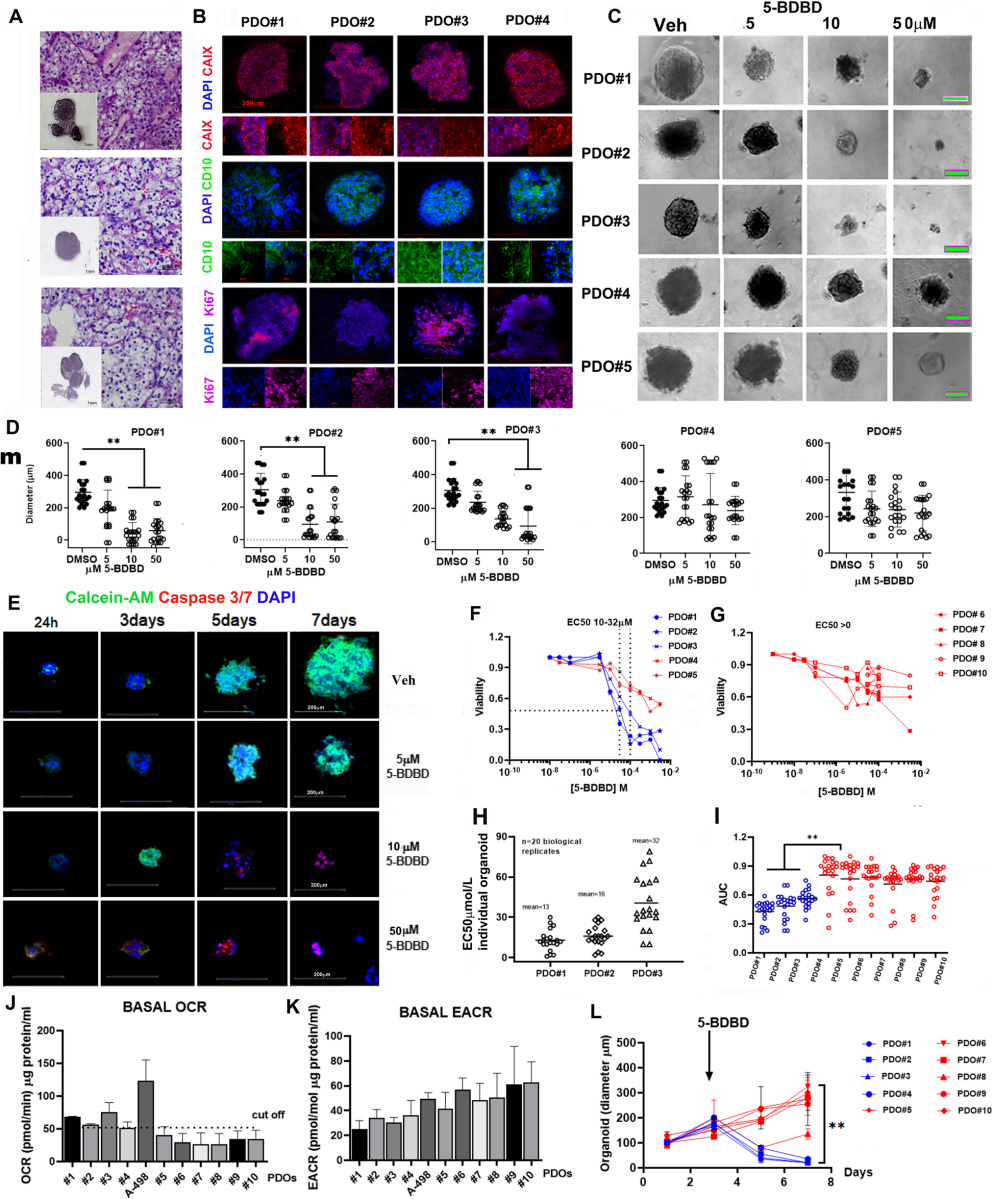
P2XR4 expression and mitochondrial respiration in ccRCC organoids. **A** Representative hematoxylin–eosin (H&E) staining of different clear cell carcinomas PDOs sections together with the bright-field microscopy images of corresponding PDOs (inset). Scale bars = 50 μm (section) and inset scale bar = 1 mm. **B** Representative immunofluorescence images of PDOs stained with typical markers of clear cell renal cancers, e.g., CD10 (green) and carbonic anhydrase 9 (red) Ki67 (pink). Nuclei were stained with 4′,6-diamidino-2-phenylindole (DAPI) (blue). Scale bars = 100 μm or 20 μm. **C** Phase contrast images of organoids from different patients treated with different doses of 5-BDBD as indicated, or vehicle for 4 days, Scale bars = 100 μm. **D** Quantification of PDOs diameters from different patients (PDOs 1–5) treated for 4 days with the indicated dose of 5-BDBD. Data are reported as mean of 20 replicates ± SD determined by two-tailed Mann–Whitney test. **p < 0.01.** E** Immunofluorescence of PDOs incubated with DMSO or 5 μM, 10 μM or 50 μM 5-BDBD for different days. Nuclei are indicated by DAPI staining (blue), live cells by calcein-AM (green), and dead cells by Caspase 3/7 (red). Scale bars = 200 μm. **F** and **G** Dose response curve in PDOs from patients 1 to 10 treated for 4 days with different doses of 5-BDBD as indicated. Data are reported the means of 20 biological replicates for each dose. **H** IC50 (micromol/L) of individual organoids (*n* = 20) from different patients. Data reported are the mean of 20 biological replicates. **I** Area under the curve (AUC) of individual organoids from different patients. The means of twenty replicates is indicated. Significance was calculated by two-way ANOVA **p < 0.001. **J** Oxygen consumption rate (OCR) in PDOs 1–10 or parental A-498 cells measured by Seahorse XF96 analyzer. Data are representative of 4 biological replicates. The selected cut-off of OCR is indicated by line. **K** Basal extracellular acidification rate (ECAR) in PDOs and parental A-498 cells. Data are representative of 4 biological replicates. **L** PDOs diameters after different time of treatment with 5-BDBD. Red lines represent PDOs with high mitochondrial activity, blue lines are PDOs with low mitochondrial activity. Data are reported as mean of 20 replicates and ± SD. Significance was determined by unpaired Student’s t-test. **, *p* < 0.01

The therapeutic potential of specific P2XR4 inhibitor was tested in patient-derived 3D organoids (PDO) generated from surgically resected ccRCCs patients [31]. PDOs generation was successful in 50% of patients’ biopsy samples (*n* = 10) [32]. The clinical pathological characteristics of patients are summarized in Supplementary Tables 1 and 2. Plating 4 × 10^4^ cells PDOs reached a diameter of 200–300 µm after 3–7 days (Suppl. Figure 2) and showed typical clear cell carcinoma histology and renal cancer cell markers (Fig. 7A, B). To test the effect of the P2X4 inhibitor on PDOs growth, 384-well plates were used to have 20 PDO replicates for each dose and controls, and thus to improve the robustness of statistical analysis (Suppl. Figure 3A-C). In the first experiment, 5-BDBD at different doses was added for 4 days after PDOs had grown to a diameter of 200µm. The schematic representation of the experiment is reported in Suppl. Figure 3. Even though PDOsreplicates from the same patient showed some differences in their response to 5-BDBD treatment (Fig. 7 C,D), the mean data from the 20 replicates in each group indicated that 5-BDBD decreased PDOs size in a statistically significant dose-dependent manner in 3 of 10 patients (PDOs #1–3 in Fig. 7 D). Moreover, the staining with calcein-AM (as a vital stain) and caspase 3 as an apoptotic marker, of PDOs drug treated indicated that 5-BDBD not only inhibited the growth, but also induced cell death, as evidenced in a representative image (Fig. 7E). Dose–response curves in a viability experiment again showed that three PDOs were more sensitive to treatment that the rest, with a mean of IC_50_ ranging from 10 to 32 μM after 4 days of treatment (Fig. 7F,G) although the IC_50_ of individual biological replicates was different (Fig. 7H). Furthermore, area under the curve (AUC) of all PDOs confirmed the significant difference between PDOs #1–3 and the others by two-way ANOVA (Fig. 7H). In contrast, all PDOs displayed similar response patterns to everolimus and AZD8055 (Suppl. Figure 4). The consistency of the results in replicates of the same treatment suggests that the responses of PDOs generated from the same patient were stable and confirmed their suitability as a model for testing drug sensitivity (Fig. 7H, I). Because 5-BDBD inhibitory activity was greater in cells displaying pronounced mitochondrial activity (Fig. 6A), we wondered whether this effect would have observed in PDOs. To test this hypothesis, we evaluated the mitochondrial oxygen consumption rate and glycolysis in all PDOs as well as in control A-498 cells (Fig. 7J,K). We divided PDOs into two subgroups: the first subgroup with high mitochondrial activity (PDOs #1,2,3,4) with basal OCR > 50 mmol/min/µg corresponding to half value of control cells (A-498); the second group (PDOs #5–10) with low mitochondrial activity (basal OCR < 50 mmol/min/g) and variable glycolysis (Fig. 7J, K) and then analyzed variation of PDOs diameters during 5-BDBD treatment. Results indicated that three out of four PDOs with high mitochondrial activity showed significantly reduction in diameter after 4 days of 5-BDBD treatment, compared to PDOs with low mitochondrial activity (Fig. 7L). Together, these results suggest that P2X4 is a vulnerability receptor that mediates mitochondrial calcium uptake and activity.

### P2XR4 expression is a therapeutic vulnerability in vivo.

**Fig. 8:**
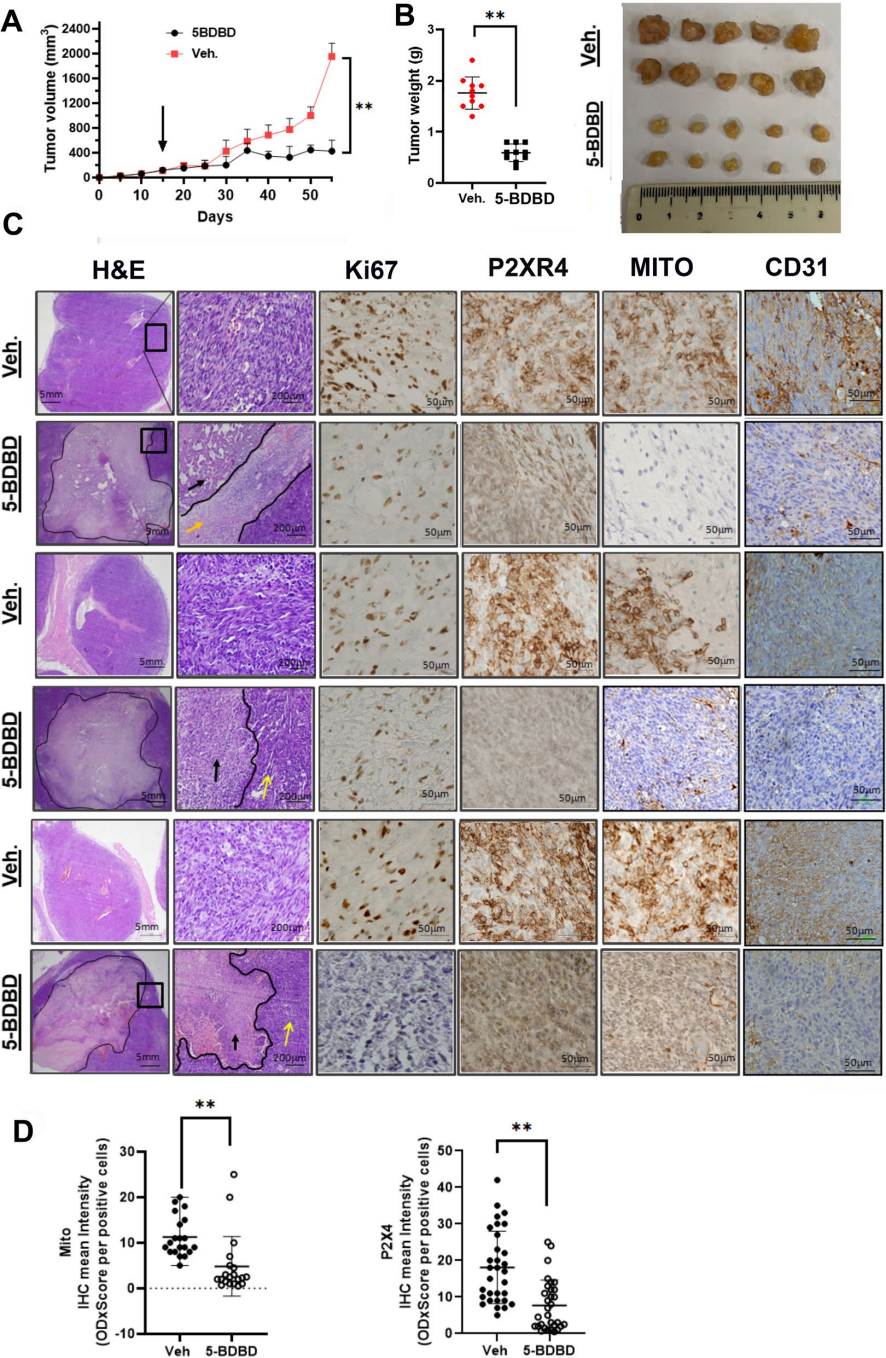
Anti-tumor effect of P2X4R inhibition in a xenograft model of ccRCC. **A** Effect of 5-BDBD treatment and vehicle control on tumor volume (growth) in mice (*n* = 5 per group). **B** Effect of 5-BDBD on tumor weight at the end of the experiment, i.e., after 40 days of treatment. **C** Representative images of tumor section from 5-BDBD and vehicle (Veh.) treated mice stained with H&E or antibodies to Ki67, P2XR4, mitochondria and CD31. Scale bars = 5 mm and 50 μm. Black line indicate necrotic area, yellow arrows inflammatory area. **D** Quantitative histoscore using QuPath of P2XR4 and mitochondria immunostaining in tumor sections of all mice (*n* = 5). **, *p* < 0.01 of for slides for each mice

To further examine the protective effect of P2XR4 on tumor growth, A-498 cells with the highest mitochondrial activity were implanted into nude mice. Beginning on day 14, when the tumors reached 50–70 mm^3^, mice were treated with vehicle control or 10 mg/kg 5-BDBD i.p. for 55 days. The tumors grew rather slowly, but the treatment effect of 5-BDBD became evident after 25 days. On day 55, the growth of the vehicle-treated tumors was more than five times greater, and they weighed 3.4 ± 4.5% more than 5-BDBD treated tumors (Fig. 8A, B). Vehicle-treated tumor sections stained with hematoxylin and eosin showed pleiotropic cellular morphology, Ki67 positive cells and the presence of mitochondria, and the P2XR4 signal localized in the cytoplasm and on membranes (Fig. 8C). In contrast, tumor sections from 5-BDBD treated mice showed large necrotic/fibrotic areas and peripheral inflammation (indicated by yellow arrows). These sections were largely Ki67, mitochondria and P2XR4 negative, except for the residual tumor areas (Fig. 8C, D). Together, these results support the hypothesis that P2X4 receptor contributes to mitochondrial metabolic activity and represents a potential therapeutic target for the treatment of renal carcinoma.

## Discussion

Our study investigated the potential role of P2XR4 in regulating mitochondrial activity in ccRCC cells in vitro*,* in a patient derived organoids model, and in vivo. Overall, results suggest that P2XR4 is a potential therapeutic target in ccRCC with high mitochondrial activity.

Cancer cells depend on lysosomes and mitochondria as energy providers and regulators of the transition to a more aggressive state [33, 34]. Following ATP stimulation, lysosomal P2XR4 releases intracellular calcium, which is necessary for promoting mitochondrial metabolism and resistance to oxidative stress and necrosis. Our studies indicate that P2XR4 is a part of the intricate network between organelles and represents a vulnerable target for treatment in ccRCC. P2XR4 was transcriptionally upregulated in the TCGA database of primary ccRCC, compared to adjacent tissues, and correlated with poor prognosis. Similarly, the expression of the P2XR4 receptor protein in the ATLAS database and our own tissue samples was weak or moderate in normal tissues, whereas tumors showed high expression. A significant positive correlation was observed between P2XR4 mRNA and proteins associated with mitochondrial activity and antioxidant mechanisms, such as superoxide dismutase 2 and glutathione peroxidase 4, in ccRCC samples from the TCGA cohort. Interestingly, our screening of renal carcinoma cell lines revealed higher mitochondrial activity in A-498 and SNC-12 cells overexpressing P2XR4 than that in other renal cancer cell lines or normal kidney cells.

P2XR4 is a member of the P2X receptor family of cation-permeable ligand-gated ion channels that open in response to ATP binding [18]. It is mainly localized on endolysosomes, but shuttles to and fuses with the cell membrane when stimulated [19, 34, 35]. It is widely expressed in the central nervous system and throughout the periphery and has many regulatory functions, including inflammatory pain, stimulation of the secretion of prostaglandin chemokines by immune cells, and motility of T cells [36–39]. Several studies have also shown a correlation between higher expression of the P2X4 receptor and lysosomal exocytosis of active hydrolases, increased cancer cell migration, survival, tumor progression [9, 40–43], and vesicular trafficking [44, 45]. P2XR4 also stimulates the growth and motility of endothelial cells and promotes neoangiogenesis and in vivo tumor growth [19]. Our present study suggests that P2XR4 regulates the interaction between lysosomes and mitochondria by modulating antioxidant metabolism and oxidative phosphorylation in cell cultures and PDOs generated from tumor biopsies of individual patients. Pharmacological and genetic disruption of P2XR4 resulted in a dose-dependent reduction in mitochondrial and glutathione activity, antioxidant response, increased lipid peroxidation, ROS formation, and cell death. Interestingly, a recent study in colon carcinoma indicated that P2XR4 inhibition increases ROS accumulation and prevents mTORC1 activity in cells sensitive to chemotherapy [46].

Our data provide the first evidence that P2XR4 is a key mediator of the interplay between lysosomes and mitochondria in tumor metabolism through Ca^2+^ homeostasis [47]. Recent data have shown that mitochondrial-lysosomal contact regulates various organelle functions, including transfer among them of different metabolites [25, 48]. We showed that inhibition of P2XR4 resulted in an overload of mitochondrial calcium and alteration of the pore membrane potential. Since the blockage of mitochondrial activity prevented P2XR4 contribution to cytoplasmic calcium, and the mechanism attributed to P2XR4 is a kiss-and-run-type fusion, in addition to full fusion [17], we can speculate that lysosomal P2XR4 provides the amount of Ca^2+^ necessary for mitochondrial pore expansion, mediating efficient transfer of Ca^2+^ into the mitochondria. Moreover, P2XR4 inhibition induces lysosomal damage, increased lipid peroxidation, and lysophagy.

Lysosomes have also been implicated in RTKI drug resistance [49]. Polymorphisms in lysosome autophagic genes are associated with poor outcomes in ccRCC [50], and mutations in SETD2, a frequent molecular feature of ccRCC, suppress autophagy regulation [51]. However, lysosomes are primarily Ca^2+^ signaling hubs that govern multiple cellular processes and the extracellular microenvironment. Furthermore, our data suggest that the pro- or anti-tumor roles of lysosomal Ca^2+^ channels can differ according to the specific genetic context, type of cancer, malignancy stage, and signals from the microenvironment [16].

Treatment of ccRCC is rapidly evolving. The current therapeutic landscape includes a combination of immune checkpoint inhibitors (ICIs) and RTKI. However, the development of drug resistance remains a major challenge. The development of therapeutic strategies targeting tumor metabolism in ccRCC is a rational approach. Ongoing experiments in our laboratories are testing different combination therapies with RTKI, mTOR inhibitors, and P2X4 inhibitors. The results of these studies will guide future development of this novel therapeutic strategy. We recognize that the potential use of organoids in predicting responses to treatment with a P2XR4 antagonist will require additional work to validate the initial observation, including similar experiments by utilizing specimens from metastatic sites.

## Conclusion

In conclusion, our data indicate that therapies targeting mitochondrial-lysosomal interactions, such as inhibitors of P2XR4, may be an effective treatment for ccRCC, but their efficacy may depend on high mitochondrial activity. In the absence of clinical assays for measuring mitochondrial activity, patient-derived organoids may offer a predictive model for P2XR4 directed therapies.

## Supplementary Information

**Additional file 1: Suppl. Figure 1.** P2X4 in clear cell renal carcinoma correlates with mitochondrial antioxidant proteins by mRNA seq data bases. **Suppl. Figure 2.** (A) Representative MRI image of a patient with kidney carcinoma. (B) Organoid diameters after different time of culture. Data represent 3 different measurements of 3-5 biological replicates. Scale bars = 50 μm (**C**) Representative hematoxylin-eosin (H&E) staining of different clear cell carcinomas biopsies together with the bright-field microscope images of corresponding H&E stained ccRCC organoids (inset). Scale bars = 50 μm. **Suppl. Figure 3**. (A) Representative image of organoids treated with DMSO vehicle or 5-BDBD for different times and then stained with Calcein-AM (green) indicating vital cells or PI (red) indicating necrotic ones. Scale bar = 100 µm. (B) Concentration response to 5-BDBD determined in 3D culture assay measured at day 10 yielding an IC50 value of 7.556 µM (C) Schematic representation of plate view software dosage of 5-BDBD tested on 20 replicates for individual dose in single patient analyzed by CELIGO software. (D) Comparison of organoids growth for 3 days than treated with DMSO or 5-BDBD (5µM) for additional 7 days. Scale bar 100µm (E) Colony formation assay. The number of colony formed by A-498 cells 10^5^ per well in presence of Vehicle (DMSO) or 5-BDBD at 5μM were cultured for 14 days colonies were stained with crystal violet and then photographed. **Suppl. Figure 4**. (A) Phase contrast image of representative organoids from different patients (PDO#1-5) treated with different doses of Everolimus for 7 days. (B,C) Quantification by MTT assay of the vitality reported as ratio to non treated organoids x100 from different patients (PDO#1-5) treated for 7 days with increasing doses of Everolimus or EZD8055, as indicated. Data are means of 20 replicate organoids from the same patient for each dose. Error bars are SD of 29 biological replicated for each dose. **Table 1.** Clinical features of the Clear Cell Renal Carcinomas. **Table 2. **Imaging characteristics of renal lesions.

## Data Availability

All data generated or analyzed during this study are included in this published article and its supplementary information files, except for the data of the publicly accessible large databases.

## Abbreviations

PDO: Patient derived organoids
KIRC: Kidney renal clear cell carcinoma
ccRCC: Clear cell renal cell carcinoma
RTKI: Receptor tyrosine kinase inhibitor
P2XR4: Purinergic receptor X 4
P2XR7: Purinergic receptor X 7
ROS: Reactive oxygen species
IP3Ki: Inositol 1,4,5-trisphosphate receptor inhibitor
OCR: Oxygen consumption rate
ECAR: Extracellular acidification rate
