# Supplementary material for: Therapeutic targeting of P2X4 receptor and mitochondrial metabolism in clear cell renal carcinoma models
Source: J Exp Clin Cancer Res. 2023 May 26;42:134. doi: 10.1186/s13046-023-02713-1 (PMC10214673; doi:10.1186/s13046-023-02713-1)
Supplement: Supplementary file 1 — Additional file 1: Suppl. Figure 1. P2X4 in clear cell renal carcinoma correlates with mitochondrial antioxidant proteins by mRNA seq data bases. Suppl. Figure 2. (A) Representative MRI image of a patient with kidney carcinoma. (B) Organoid diameters after different time of culture. Data represent 3 different measurements of 3-5 biological replicates. Scale bars = 50 μm (C) Representative hematoxylin-eosin (H&E) staining of different clear cell carcinomas biopsies together with the bright-field microscope images of corresponding H&E stained ccRCC organoids (inset). Scale bars = 50 μm. Suppl. Figure 3. (A) Representative image of organoids treated with DMSO vehicle or 5-BDBD for different times and then stained with Calcein-AM (green) indicating vital cells or PI (red) indicating necrotic ones. Scale bar = 100 µm. (B) Concentration response to 5-BDBD determined in 3D culture assay measured at day 10 yielding an IC50 value of 7.556 µM (C) Schematic representation of plate view software dosage of 5-BDBD tested on 20 replicates for individual dose in single patient analyzed by CELIGO software. (D) Comparison of organoids growth for 3 days than treated with DMSO or 5-BDBD (5µM) for additional 7 days. Scale bar 100µm (E) Colony formation assay. The number of colony formed by A-498 cells 105 per well in presence of Vehicle (DMSO) or 5-BDBD at 5μM were cultured for 14 days colonies were stained with crystal violet and then photographed. Suppl. Figure 4. (A) Phase contrast image of representative organoids from different patients (PDO#1-5) treated with different doses of Everolimus for 7 days. (B,C) Quantification by MTT assay of the vitality reported as ratio to non treated organoids x100 from different patients (PDO#1-5) treated for 7 days with increasing doses of Everolimus or EZD8055, as indicated. Data are means of 20 replicate organoids from the same patient for each dose. Error bars are SD of 29 biological replicated for each dose. Table 1. Clinical features of the Cl [file 13046_2023_2713_MOESM1_ESM.docx]

**Supplementary materials**

**Therapeutic targeting of P2X4 receptor and mitochondrial metabolism in clear cell renal carcinoma**

Christofer Rupert^1^, Carmela Dell’ Aversana^2,3^, Laura Mosca^3^, Vittorino Montanaro^4^, Davide Arcaniolo^5^, Marco De Sio^5^, Antonio Bilancio^3^, Lucia Altucci^2,3,6^, Wulf Palinski^7^, Roberto Pili^1#^, Filomena de Nigris^3#^

1 Division of Hematology and Oncology, Jacobs School of Medicine and Biomedical Sciences, University at Buffalo, Buffalo, NY

2 Institute of Experimental Endocrinology and Oncology Gaetano Salvatore (IEOS)-CNR, Naples, IT

3 Department of Precision Medicine, University of Campania L. Vanvitelli, Naples, IT

4 Urology Unit, San Leonardo Hospital, Castellammare di Stabia, Naples, IT

5 Department of General and Specialistic Surgery, University of Campania L. Vanvitelli, Naples, IT

6 BIOGEM, Ariano Irpino, Avellino, IT

7 Department of Medicine, University of California San Diego, La Jolla, CA

#Corresponding authors:# [rpili@buffalo.edu](mailto:rpili@buffalo.edu); [Filomena.DENIGRIS@unicampania.it](mailto:Filomena.DENIGRIS@unicampania.it)


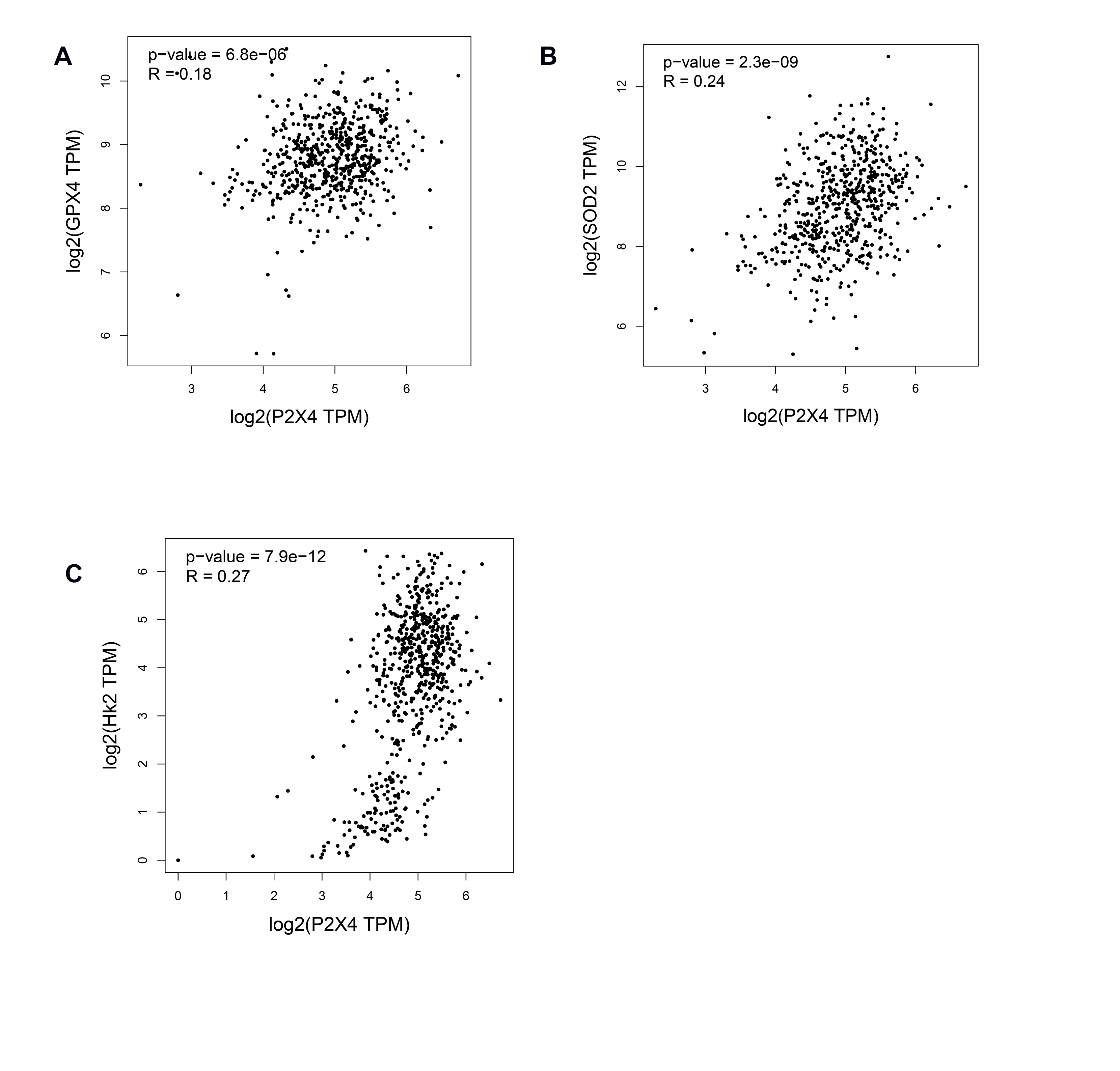


**Suppl.**  **Figure 1. P2X4 in clear cell renal carcinoma correlates with mitochondrial antioxidant proteins by mRNA seq data bases.** Pearson’s correlation between expression of the P2X4 gene and (**A**), GPX4 (p = 7e^-6^) (**B**), SOD2 (p = 2e^-.9^) and(**C**). HK2 (p = 7e^-12^) mRNAs in KIRC samples using GEPIA data base. Positive correlations of P2X4 were established with.

Abbreviations P2XR4 (Purinergic receptor X4); SOD2, Superoxide dismutase 2, mitochondrial HK2 (Hexokinase 2); GPX4,(Glutathione peroxidase 4.


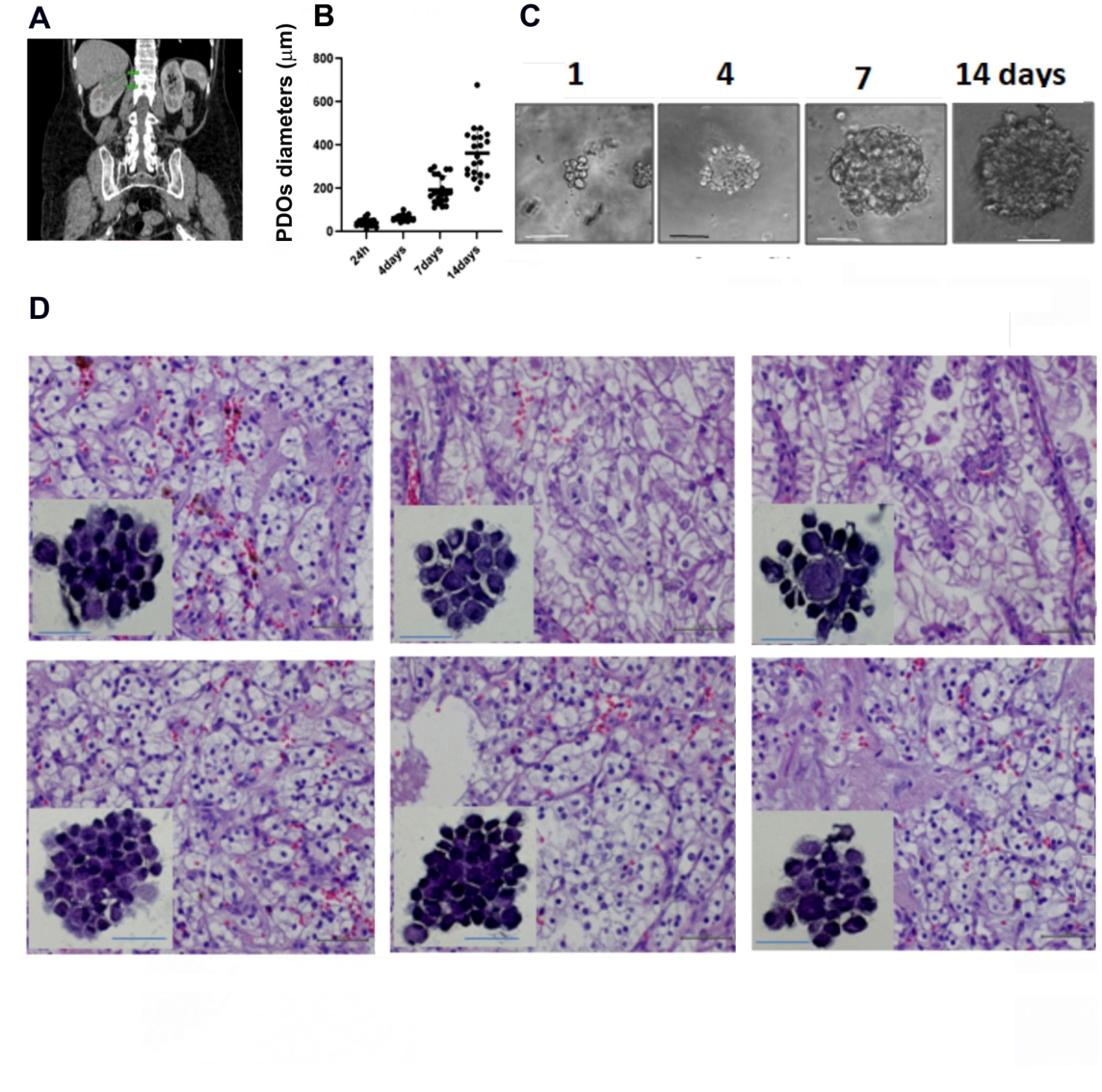


**Suppl. Figure 2**. (**A**) Representative MRI image of a patient with kidney carcinoma. (**B**) Organoid diameters after different time of culture. Data represent 3 different measurements of 3-5 biological replicates. Scale bars = 50 μm~~.~~ (**C**) Representative hematoxylin-eosin (H&E) staining of different clear cell carcinomas biopsies together with the bright-field microscope images of corresponding H&E stained ccRCC organoids (inset). Scale bars = 50 μm.


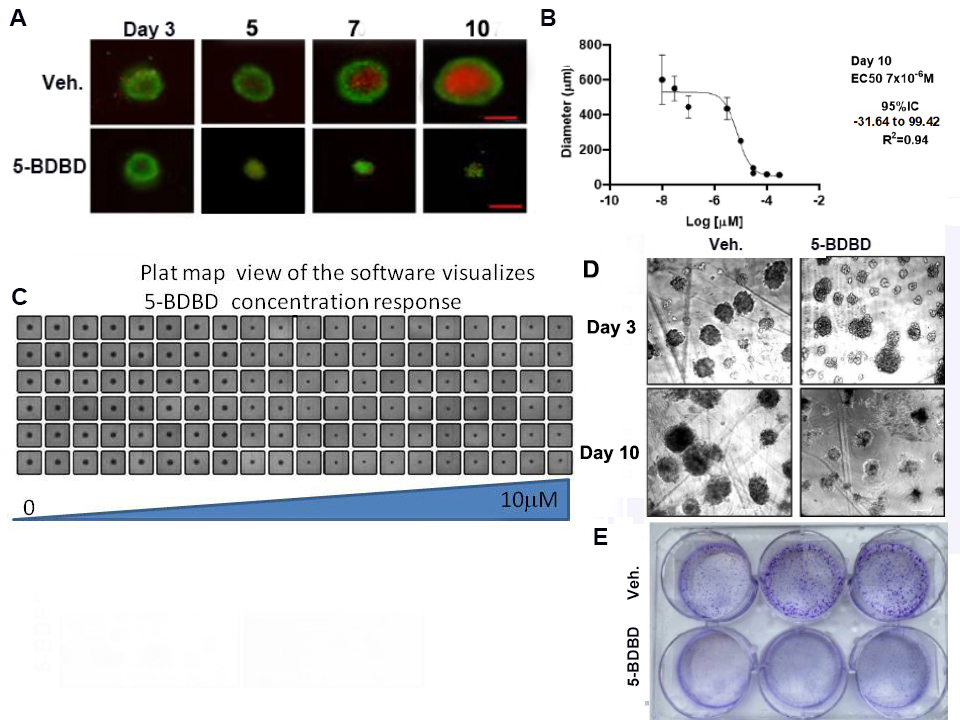


**Suppl. Figure 3**. (**A**) Representative image of organoids treated with DMSO vehicle or 5-BDBD for different times and then stained with Calcein-AM (green) indicating vital cells or PI (red) indicating necrotic ones. Scale bar = 100 μm. (B) Concentration response to 5-BDBD determined in 3D culture assay measured at day 10 yielding an IC50 value of 7.556 µM (**C**) Schematic representation of plate view software dosage of 5-BDBD tested on 20 replicates for individual dose in single patient analyzed by CELIGO software. (**D**) Comparison of organoids growth for 3 days than treated with DMSO or 5-BDBD (5μM) for additional 7 days. Scale bar 100μm **(E)** Colony formation assay. The number of colony formed by A-498 cells 10^5^ per well in presence of Vehicle (DMSO) or 5-BDBD at 5μM were cultured for 14 days colonies were stained with crystal violet and then photographed


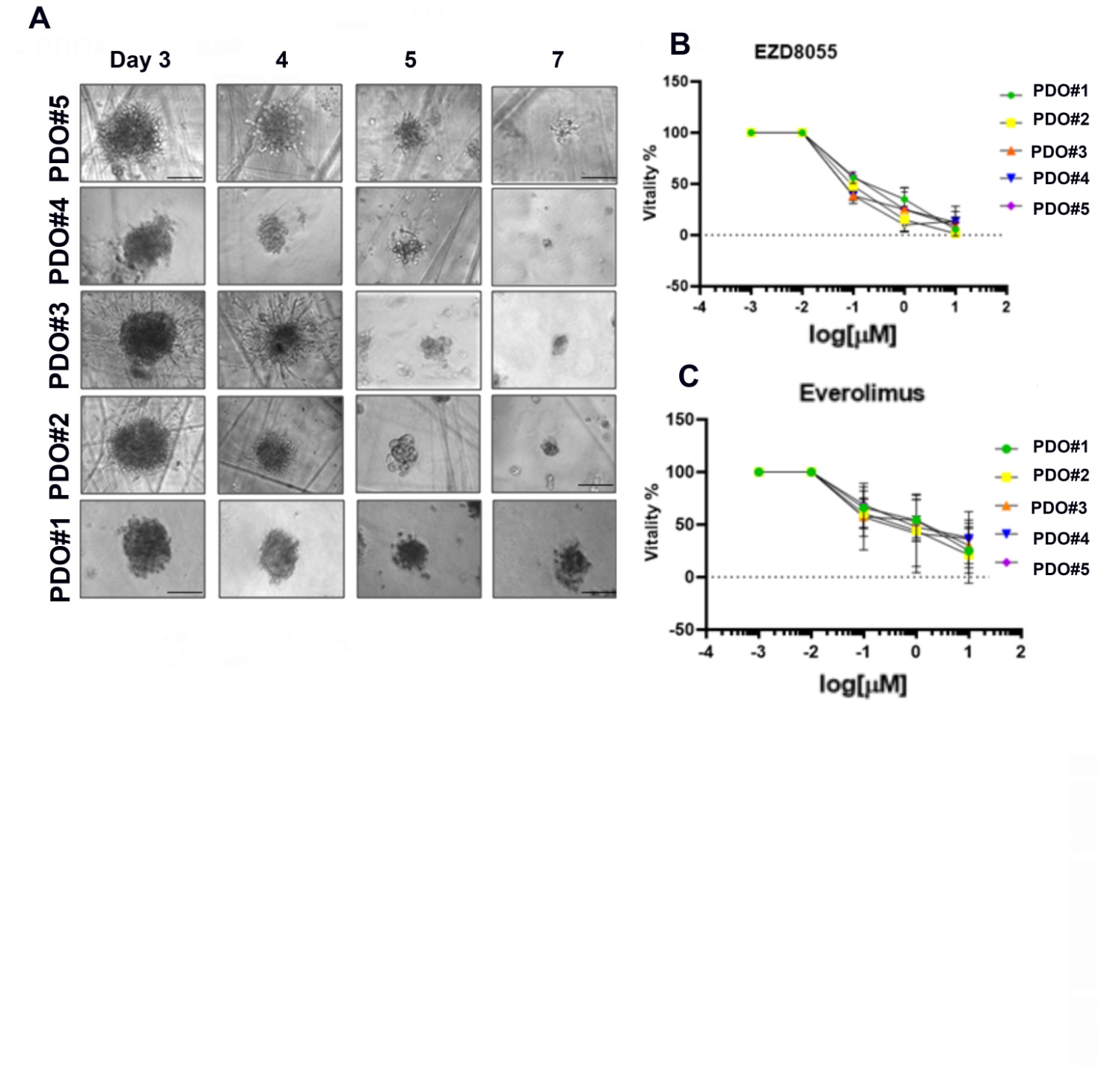


**Suppl. Figure 4**. (**A**) Phase contrast image of representative organoids from different patients (PDO#1-5) treated with different doses of Everolimus for 7 days. (**B,C**) Quantification by MTT assay of the vitality reported as ratio to non treated organoids x100 from different patients (PDO#1-5) treated for 7 days with increasing doses of Everolimus or EZD8055, as indicated. Data are means of 20 replicate organoids from the same patient for each dose. Error bars are SD of 29 biological replicated for each dose.

Supplementary material to culture organoids

| Advanced DMEM/F-12 | Thermo Fisher Scientific | 12634-010 | 1x |
| --- | --- | --- | --- |
| Antibiotic-Antimycotic | Thermo Fisher Scientific | 15240-062 | 1x |
| GlutaMAX™ Supplement | Thermo Fisher Scientific | 35050-061 | 1x |
| HEPES | Thermo Fisher Scientific | 15630-080 | 10 mM |
| B-27™ Supplement (50X),  serum free | Gibco | 17504-044 | 1x |
| N-Acetylcysteine | Sigma | A9165 | 1.25 mM |
| Nicotinamide | Sigma | N0636 | 10 mM |
| SB202190 | Sigma | S7076 | 10 mM |
| Y-27632 | Abmole Bioscience | M1817 | 10 mM |
| Human EGF | Peprotech | AF-100-15 | 50 ng/mL |

**Supplementary Table 1**

| **Table 1 Clinical features of the Clear Cell Renal Carcinomas** | | | | | | | | | | | |
| --- | --- | --- | --- | --- | --- | --- | --- | --- | --- | --- | --- |
| Case no. | **Age (years)** | Sex | **BMI (kg/m^2^)** | Laterality | **Tumor size (cm)** | **Clinical symptom** | **Creatinine (µmol/l)** | **Past medical history** | **Smoking/drinking history** | **Operative type** | **Other kidney disease** |
| 1 | 46 | M | 24.6 | R | 4.5 | Flank pain | 62 | None | Smoking history | RN | None |
| 2 | 34 | M | 20.2 | L | 3.5 | No symptom | 92 | HTN | NA | PN | None |
| 3 | 36 | M | NA | R | 4.5 | Abdominal pain | 85 | HTN | Smoking history | PN | None |
| 4 | 44 | F | 21.2 | L | 3.5 | No symptom | 101 | HTN | None | RN | None |
| 5 | 61 | M | 24.3 | L | 2.8 | No symptom | 96 | HTN | None | PN | MRC |
| 6 | 53 | M | 23.0 | L | 1.9 | Flank pain | 50 | HTN, | None | RN | None |
| 7 | 44 | M | 29.4 | L | 1.6 | No symptom | 75 | None | None | PN | None |
| 8 | 49 | M | 25.4 | L | 1.2 | Flank pain | 55 | HTN | None | PN | None |
| 9 | 42 | F | 28.7 | L | 1.2 | No symptom | 42 | None | None | PN | None |
| 10 | 60 | M | NA | L | 2.6 | No symptom | 75 | DM | Smoking history | PN | None |
| BMI, body mass index; M, man; F, female; R, right; L, left; HTN, hypertension; DM, diabetic mellitus; cerebral infarction;; PN, partial nephrectomy; RN, radical nephrectomy; NA, not available. | | | | | | | | | | | |

**Supplementary Table 2**

| **Table 2 Imaging characteristics of renal lesions** | |
| --- | --- |
| **Features** | **ccRCC (n=10)** |
| Age (year) | 54.5±10.7 |
| Lesion size (cm) | 3.5±0.6 |
| **Enhancement pattern (%)** |  |
| Homogeneous | 4 |
| Heterogeneous | 6 |
| **Tumor contour (%)** |  |
| Smooth | 8 |
| Other | 2 |
| Calcification (%) | 1 |

ccRCC clear cell renal carcinoma cells


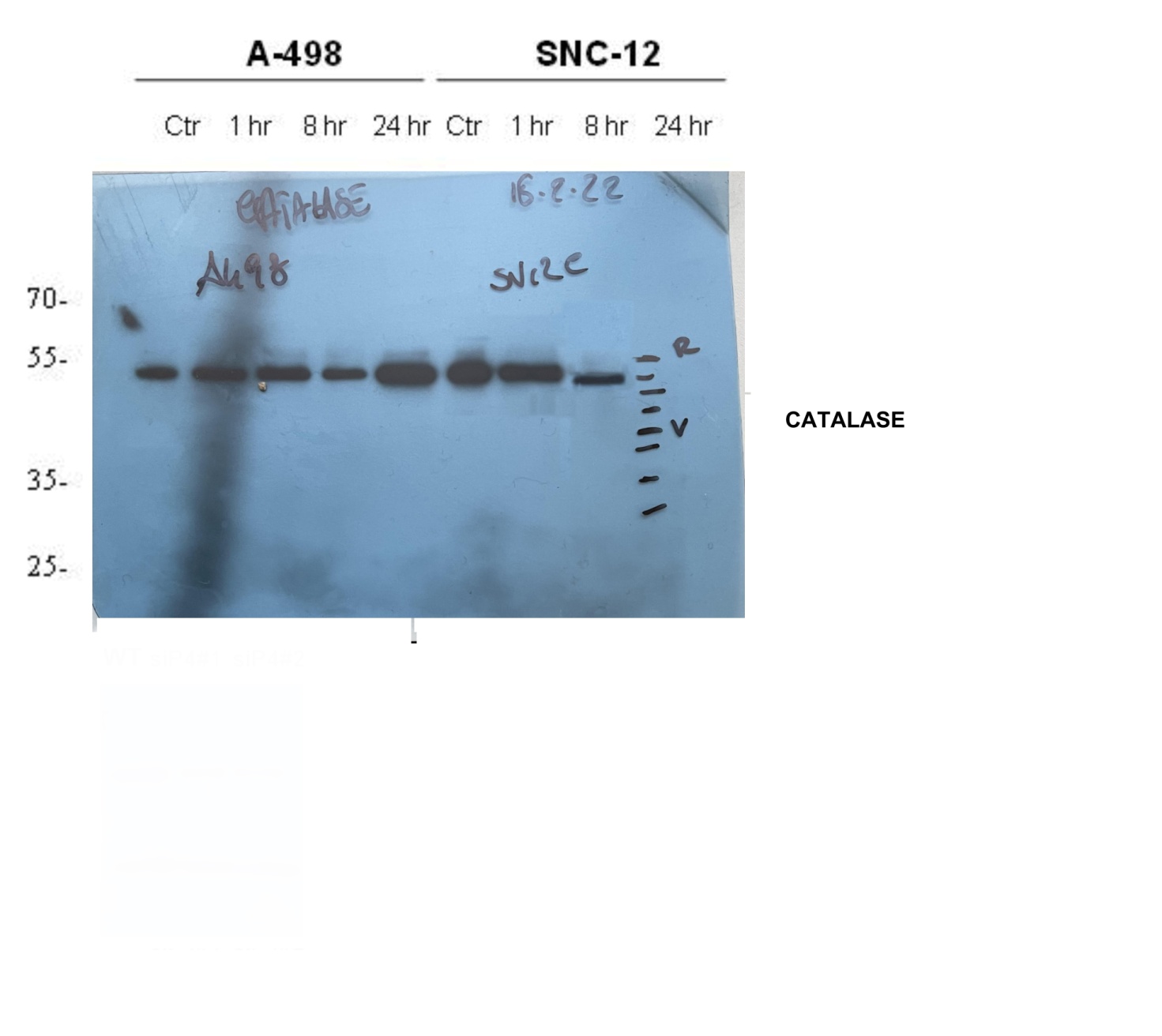


**Orgininal slide from Catalase wester blot of Figure 4 panel F** Western blot of protein extracts from A-498 and SNC-12 cells collected at different time points from 5-BDBD treatment stained with Catalase antibody.


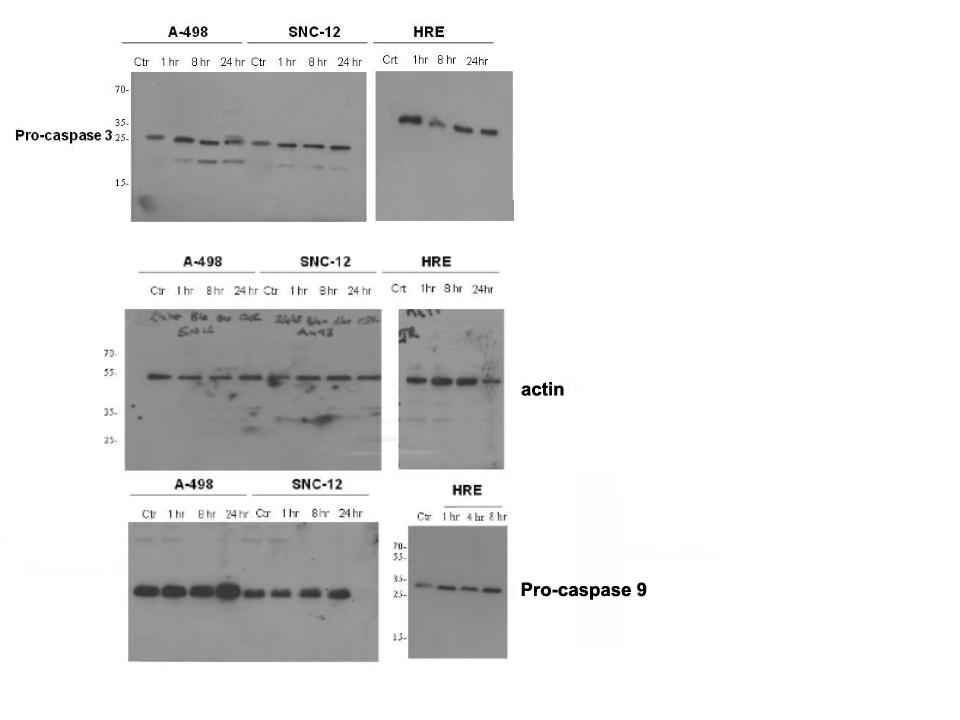


**Original slide of western blot from Figure 5 panel F**, Western blot analysis of pro-caspase 9 and 3 in protein extracts from A-498, SNC-12, and control HRE cells treated with 5-BDBD for different times. Quantitative evaluation of protein expression relative to actin.


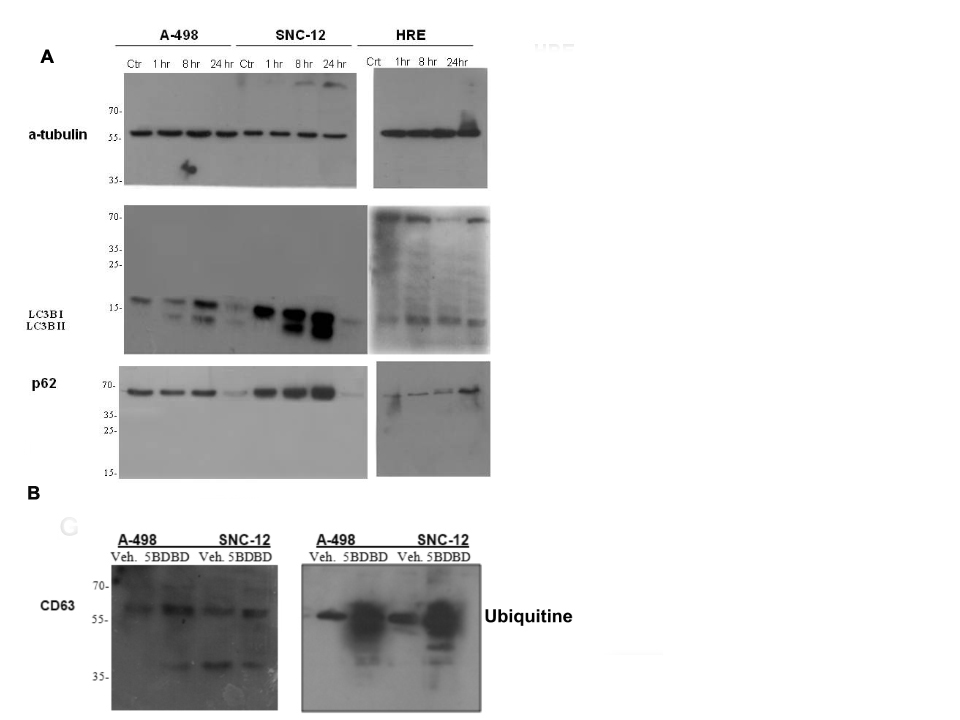


**Panel A original slides from western blot present in Figure 6 panel E reporting protein extracts from** SNC-12, A-498 and HRE cells treated or not with 5 μM 5-BDBD for different times and analyzed with LC3BI/II and p62 antibodies, as indicated. Western blot bands were normalized with tubulin. **Panel B** Western blot of extracellular microvesicles prepared from A-498 and SN-C12 cells treated with 5-BDBD and control vehicle (0.1% DMSO) immunoprecipitated with CD63 antibody (left) and Western blot analyzed with poli-Ub antibody (right)
